# Specific light-harvesting complexes mediate grana stacking and prevent energy spillover between photosystems in plant chloroplasts

**DOI:** 10.1093/plcell/koag256

**Published:** 2026-09-01

**Authors:** Zeno Guardini, Rodrigo L Gomez, Johannes Stuttmann, Parveen Akhtar, Petar H Lambrev, Luca Dall’Osto, Roberto Bassi

**Affiliations:** Dipartimento di Biotecnologie, Università di Verona, Strada Le Grazie 15, Verona 37134, Italy; Dipartimento di Biotecnologie, Università di Verona, Strada Le Grazie 15, Verona 37134, Italy; Department of Plant Genetics, Institute for Biology, Martin Luther University Halle-Wittenberg, Weinbergweg 10, Halle 06120, Germany; HUN-REN Biological Research Centre, Institute of Plant Biology, Szeged, Temesvári krt. 62, Szeged 6726, Hungary; HUN-REN Biological Research Centre, Institute of Plant Biology, Szeged, Temesvári krt. 62, Szeged 6726, Hungary; Dipartimento di Biotecnologie, Università di Verona, Strada Le Grazie 15, Verona 37134, Italy; Dipartimento di Biotecnologie, Università di Verona, Strada Le Grazie 15, Verona 37134, Italy; Accademia Nazionale dei Lincei, Palazzo Corsini, Via della Lungara 10, Rome 00165, Italy; Stazione Zoologica Anton Dohrn, Villa Comunale, Naples 80121, Italy

## Abstract

Land plant chloroplasts are characterized by a prominent morphological feature: the stacking of thylakoid membranes into grana, ie defining a continuous membranous structure surrounded by the stroma and folded into appressed grana and non-appressed domains, enclosing the thylakoid lumen.

Photosystem II and its antenna system are concentrated, whereas Photosystem I and ATPase are located in the grana end membranes and, in the stroma, membranes connecting grana. Thylakoid stacking promotes the physical separation of Photosystems I and II, thereby ensuring a balanced flow of excitation energy between them. Through a genetic dissection of the light-harvesting complex subfamily Lhcb, we showed that removal of the antenna system serving Photosystem II completely abolished grana formation, whereas deletion of specific Lhcb subgroups reduced stacking by up to 40%. Lhcb5 alone was sufficient to partially restore stacking, whereas exclusive expression of Lhcb2 resulted in grana with an unusually large size, which disassembled upon illumination. Time-resolved fluorescence measurements revealed that the extent of excitation energy spillover from PSII to Photosystem I correlated with the level of Lhcb depletion, rather than with granal architecture. These findings provide genetic evidence for a functional link between Photosystem II antenna composition, lateral heterogeneity, and excitation energy partition between photosystems.

## Introduction

Land plant chloroplast contains chlorophylls (Chl)- and carotenoids (Car)-binding complexes within its inner membranes, known as thylakoids (Gunning et al. 2006). A distinctive feature is their remarkably complex organization into structures called grana (Wietrzynski et al. 2025), which are characteristic of land plant species (Iwai et al. 2018). Grana consist of stacks of round, flattened vesicles connected by pairs of unstacked membranes, referred to as stromal membranes (Kirchhoff 2019). Membrane stacks are also present in green algae; however, they lack the distinctive regular organization found in higher plants (Olive and Vallon 1991).

Freeze-etching electron microscopy (EM) combined with mutant analysis has shown that photosynthetic pigment–protein complexes in thylakoids are compartmentalized: Photosystem (PS) II particles are located in the appressed region of the grana (Armond and Arntzen 1977), whereas PSI and ATPase complexes are hosted in the stromal membranes (Armond et al. 1977). The cytochrome (Cyt) *b*_6_*f* complex is evenly distributed between the stromal regions and the grana margins (Vallon et al. 1991). Grana of vascular plant chloroplasts are a successful evolutionary innovation (Mullineaux 2005) and are ubiquitously present in vascular plants, suggesting that they play critical roles in optimizing photosynthetic performance and enabling acclimation to ever-fluctuating light environments (Chow et al. 2005; Goss et al. 2007). The tightly appressed arrangement of granal thylakoid membranes enhances light harvesting by increasing the membrane area-to-volume ratio and by enabling the functional connectivity of multiple PSIIs with large antenna sizes (Dekker and Boekema 2005). Grana also enforces the compartmentalization of PSI vs. PSII (Anderson and Melis 1983; Vallon et al. 1991), which is thought to prevent excitation energy spillover from PSII to PSI, owing to low-energy Chl forms PSI-LHCI (Murata 1969).

Because photosynthetic electron transport proceeds linearly between the 2 PSs, diffusion of plastoquinone (PQ) toward the Cyt *b*_6_*f* is hindered by PSII crowding within the grana partitions (Lavergne and Joliot 1991), thereby limiting the rate of linear electron flow (LEF). Diffusion constraints also affect PSII damaged by photoinhibition, as it needs to reach the stroma-exposed domains for repair (Mattoo et al. 1999). Consequently, the relative sizes of granal and stromal domains might be a compromise between the advantage of ensuring adequate excitation energy delivery to both PSs and the disadvantage of restricted PQ diffusion, limited long-range electron transport by plastocyanin (Höhner et al. 2020) and reduced PSII repair efficiency.

This view is supported by observations that PQ overreduction activates STN-7 and −8 kinases, which phosphorylate the trimeric antenna LHCII and the PSII core, respectively. These events lead to a reduction in granal diameter (Flannery et al. 2021) and promote the migration of a fraction of LHCII to PSI-LHCI (Goldschmidt-Clermont and Bassi 2015). It has been proposed that grana shrinkage increases the proportion of Cyt *b*_6_*f* complexes on the stroma-exposed membranes and enhances the dynamics of cyclic electron flow (CEF) and ATP synthesis, thereby helping to attain the NADPH/ATP ratio required for CO_2_ fixation (Kramer and Evans 2011).

The extent of stacking is strongly influenced by the light environment, and it has been proposed that grana dynamics enhance light harvesting under limiting light by promoting the formation of PSII-LHCII semi-crystalline arrays (Kouřil et al. 2013). Membrane stacks became organized into “true grana” by CURT1 proteins, which localize to grana margins and induce membrane curvature (Armbruster et al. 2013; Pribil et al. 2014). In the absence of CURT1 proteins, grana comprise fewer but wider membrane layers. However, the identity and mechanism of the adhesion factor(s) responsible for membrane stacking remains unresolved.

Reverse genetics and EM analysis have produced controversial results. Plants grown under intermittent light become LHCII-deficient and showed reduced grana (Argyroudi-Akoyunoglou et al. 1971; Armond et al. 1976). In contrast, the *chlorina f2* barley mutant, which lacks Chl *b* and is therefore depleted in LHCs, still maintains extensive grana stacks and preserves PSs lateral heterogeneity (Bassi et al. 1985; Kim et al. 2009). Similarly, grana are retained in the *viridis115* barley mutant, which lacks the PSII core complex, as well as in the *viridis 115* x *chlorina f2* double mutant (Simpson et al. 1989). These findings suggest that neither of the 2 major thylakoid components of the grana partitions (LHCII and PSII core) is strictly required for thylakoid cohesion. Conversely, plants lacking the PSII core complex have been reported to display increased stacking (Campoli et al. 2009; Belgio et al. 2014). Moreover, mutant plants, in which either the accumulation or the organization of LHC is perturbed, exhibit altered grana architecture (Andersson et al. 2003; Kim et al. 2009; Cui et al. 2011; Yokoyama et al. 2016). Grana were also present in mutants depleted in specific LHCII isoforms (Pietrzykowska et al. 2014; Vayghan et al. 2022), leaving the specific contribution of LHCII to grana formation unresolved.

In this study, we performed a reverse genetics analysis of thylakoid membrane stacking by combining genome editing (GE) with ultrastructure characterization, focusing on LHCs. Our results indicate that specific components of the PSII antenna system play distinct roles in thylakoid stacking. Time-resolved fluorescence spectroscopy of mutants with varying degrees of stacking further indicates that the extent of excitation energy spillover from PSII to PSI is more tightly linked to the Lhcb content than to the overall architecture of grana-stroma assemblies.

## Results

### Construction of *Arabidopsis* genotypes with selectively reduced PSII antenna system by genome editing

The construction of *Arabidopsis thaliana* genotypes with altered Lhcb protein composition was based on 2 knock-out (ko) lines: *koLhcb3,* missing the Lhcb3 component of the major LHCII complex, and the *NoM koLhcb3* genotype, devoid of the monomeric antennae Lhcb4, Lhcb5 and Lhcb6 (Dall’Osto et al. 2017), in addition to Lhcb3. A CRISPR-Cas9 mutagenesis strategy targeted each of the 5 *Lhcb1* and 3 *Lhcb2* genes (Figure S1a). This approach led to the creation of either the *koLHCII* lines (lacking all LHCII but retaining monomeric Lhcb4-6 proteins), or the *koLhcb* lines, missing both monomeric and trimeric LHCs of PSII. The *lowLHCII* genotype was selected from partial gene edits of *Lhcb1* and *Lhcb2*, in the *NoM koLhcb3* background, resulting in lines with one LHCII trimer per monomeric PSII core complex (Guardini et al. 2025). *NoM koLhcb1 koLhcb3* lines (*Lhcb2-only*) retained Lhcb2 as the sole PSII antenna (Ordon et al. 2020) (Figures S1b, c, S2).

### Growth phenotype of LHC-depleted plants

**Figure 1:**
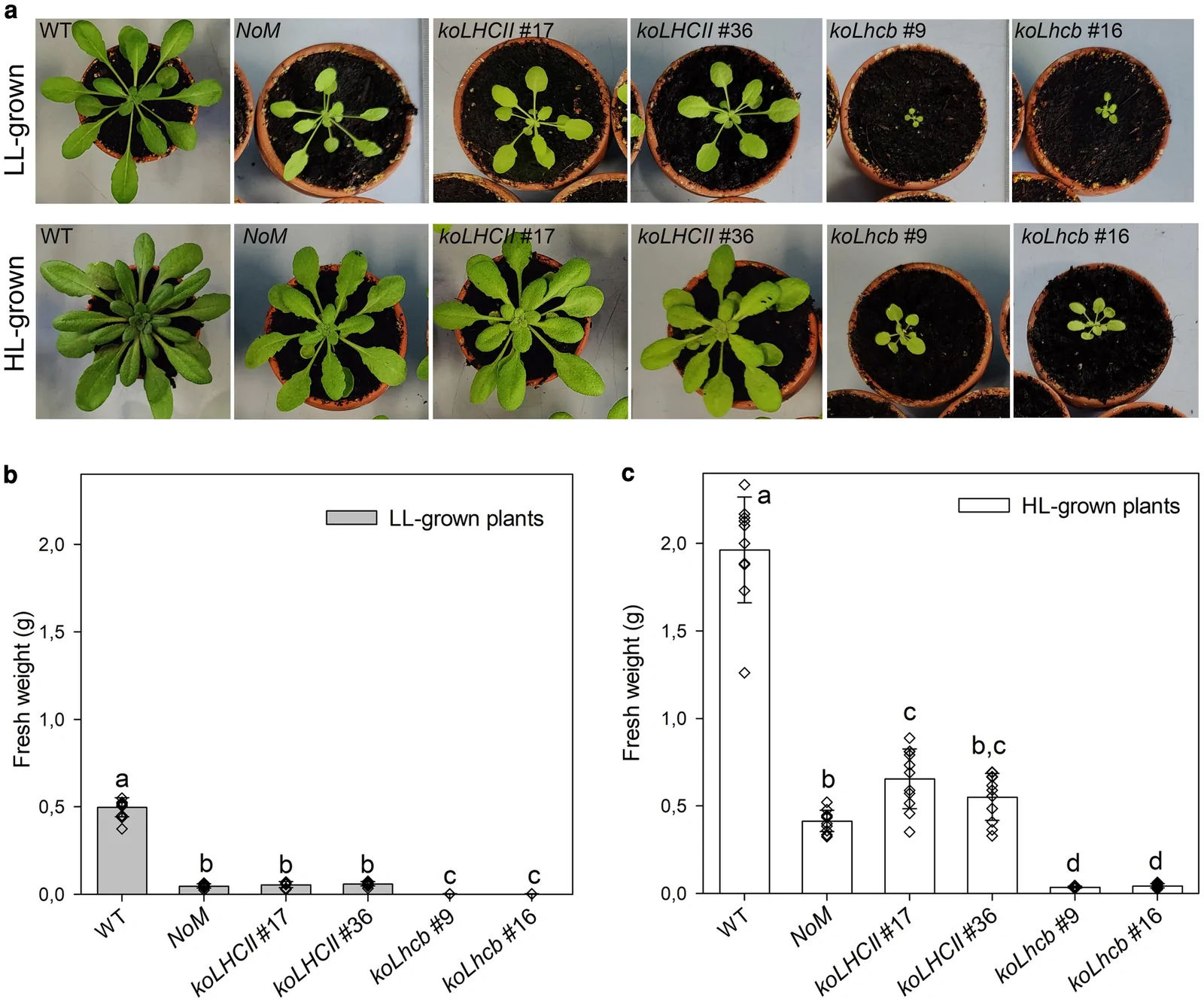
Phenotype of wild type and mutant plants. (a) Plants were grown for 6 wk under 2 different light conditions, 120 µmol photons m^−2^ s^−1^ at 23 °C, with an 8/16 h light/dark cycle (LL, upper panel), and 400 µmol photons m^−2^ s^−1^ at 23 °C, with the same light/dark cycle (HL, lower panel). (b, c**)** Fresh weight measurements for all plants, after 6 wk of growth in either LL (b) or HL (c). Values are expressed as mean ± SD, *n* ≥ 10, all individual data points are shown. Statistically significant differences from the wild type (determined via ANOVA followed by Tukey's post-hoc test at a significance level of *P* < 0.05) are indicated with different letters. Each experiment was independently replicated twice, yielding similar results.

Figure 1a presents plants grown for 6 wk under light-limiting (LL-grown) conditions (120 µmol photons m^−2^ s^−1^, 8/16 h photoperiod, 23 °C). The wild type exhibited the best growth, while the *NoM* and 2 *koLHCII* lines achieved 10% to 12% of the wild type's biomass. Meanwhile, the *koLhcb* lines grew less than 1% compared to the wild type (Fig. 1b). The pigment composition of these genotypes reflected the abundance of their pigment-protein complexes (see Figure S3 for details). The variation in growth among antenna mutants might stem from restricted photon harvesting (Figure S4) or impaired photosynthetic electron transport. To test this, plants were also grown at moderately high light (HL, 400 µmol photons m^−2^ s^−1^, 8/16 h photoperiod, 23 °C, see Fig. 1a). Under HL, the growth rate of *NoM* and *koLHCII* increased significantly, whereas it remained much slower *in koLhcb*, only slightly faster growing than in LL conditions (Fig. 1c, Figure S5). None of the lines showed obvious photosensitivity under these growth conditions: when comparing photosynthetic performance under LL vs. HL conditions, all mutants showed a decrease in both the fraction of oxidized PSII (qL) and the PSII photochemical efficiency (ϕ_PSII_) similar to those measured in the wild type, indicating that photoprotection was not significantly reduced (Table S1). Thus, increased irradiance did not fully compensate for the reduced growth observed under limiting-light conditions, suggesting that neither insufficient photon harvesting alone nor photoinhibition was the primary factor underlying growth differences among genotypes.

### Thylakoid membranes organization

**Figure 2.**
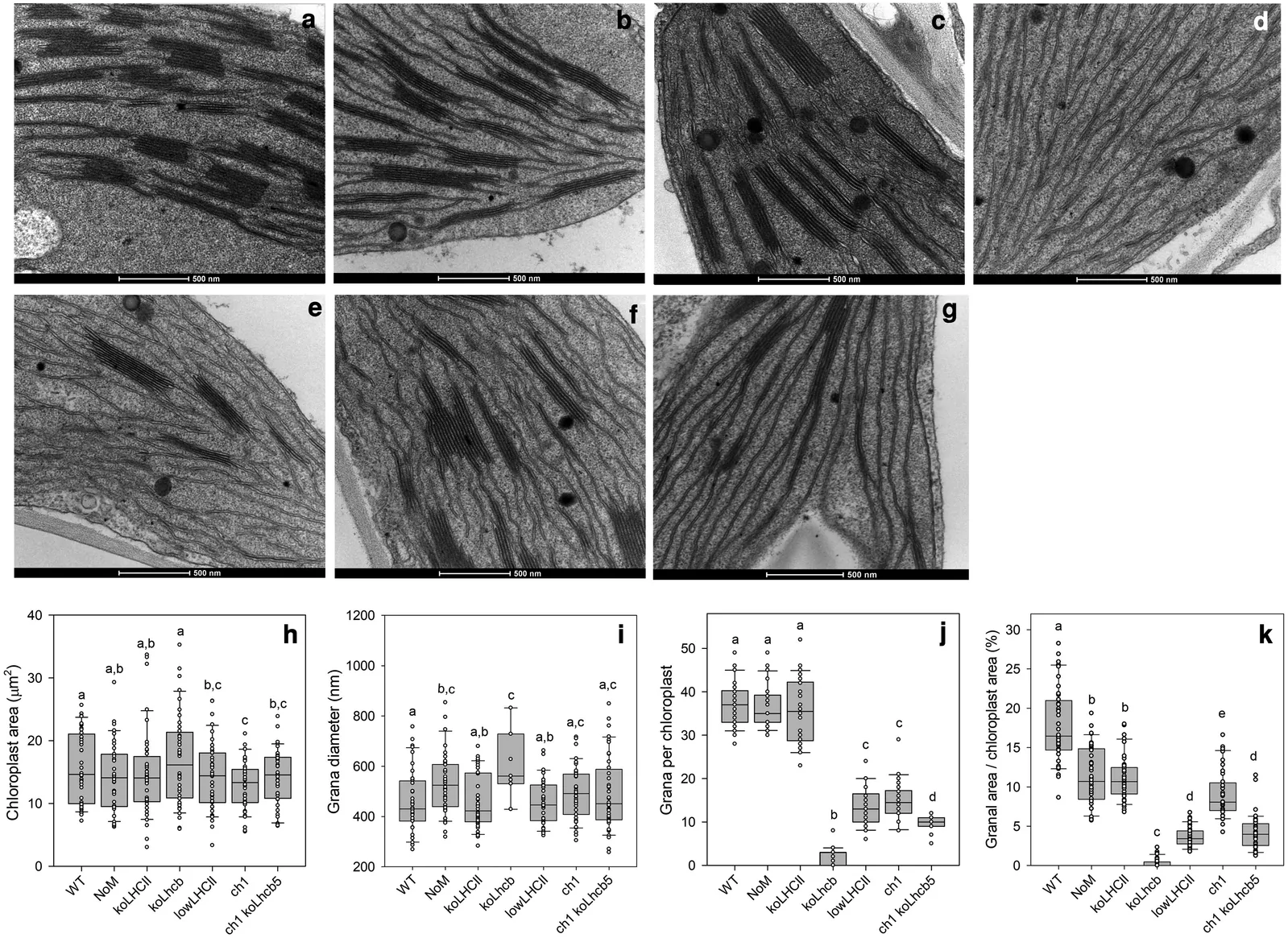
Transmission electron micrographs of plastids from leaf mesophyll cells in wild type and mutant lines. (a-d) Plants grown under short-day conditions were dark-adapted for 2 hours before harvesting their leaves. The samples were then fixed, embedded, and examined in thin sections at various magnifications. The wild type (a), *NoM* (b) and *koLHCII* (c) exhibited a characteristic organization of stroma lamellae interconnecting grana, while the chloroplasts of *koLhcb* (d) were devoid of grana. (e-g) Micrograph of *lowLHCII* (e), *ch1* (f) and *ch1 koLhcb5* (g) chloroplasts. (h-k) Statistical analysis of the extent of thylakoid stacking in wild type, LHC KO lines and Chl *b*-less mutants *ch1* and *ch1 koLhcb5.* The box plots report the following: (h) chloroplast area, measured from individual chloroplasts [n = 122 (wild type, *ch1 koLhcb5*), 124 (*NoM, koLHCII*), 127 (*koLhcb*), 132 (*lowLHCII, ch1*)]; (i) grana diameter, measured from individual grana [n = 60 (wild type, *lowLHCII*, *NoM*, *koLHCII*, *ch1*, *ch1 koLhcb5*), 7 (*koLhcb*)]; (j) grana per chloroplast, measured from individual chloroplasts (n = 30); (k) granal area *vs.* chloroplast area, measured from individual micrographs [(n = 41 (wild type, *koLhcb*), 44 (*ch1, ch1 koLhcb5*), 47 (*NoM*, *lowLHCII*), 50 (*koLHCII*)]. All individual data points are shown; in the box plots, the boxes indicate the interquartile areas, the horizontal lines show the medians, the whiskers are drawn down to the 5th percentile and up to the 95th. Values that are statistically significant (ANOVA followed by Tukey's post-hoc test at a significance level of P < 0.05) are indicated with different letters. All experiments were independently repeated twice, yielding similar results.

Antenna proteins of PSII are the most abundant components of photosynthetic membranes, suggesting that thylakoid architecture are likely to be affected in LHC-less genotypes. Therefore, we analyzed chloroplast organization by transmission EM (Fig. 2a–g). All genotypes had chloroplasts of similar size (Fig. 2h). Grana stacks were defined as being composed by at least three stacked thylakoids lamellae (Gügel and Soll 2017). Grana diameter was approximately 450 nm across genotypes, with *koLhcb* being the exception, showing a slightly larger grana diameter of about 600 nm (Fig. 2i). Note the special case of the *Lhcb2-only* genotype below.

The number of grana per chloroplast was essentially the same in wild type, *NoM* and *koLHCII*, while decreased significantly in the other mutant lines, with the most dramatic decrease detected for *koLhcb* (Fig. 2j, Figure S6). The granal cross-sectional area was measured as percentage of the chloroplast area (Fig. 2k). In wild type chloroplasts, grana occupied about 18% of the section area, consistent with a previous report (Kim et al. 2009), while this value decreased significantly (by∼30 to 40%) in both *koLHCII* and *NoM*., *koLhcb* showed a near-total depletion of grana (<1%, Fig. 2k), while *lowLHCII* had a slight recovery to 3%.

Noteworthy, *koLHCII* and *NoM* retained similar granal cross-section (Fig. 2k), despite their different LHC/PSII core stoichiometry: *koLHCII* had, on average, 3 LHC monomers per PSII core, while *NoM* had 18, ie 6 LHCII trimers per PSII core, thus 60% more than the wild type (Dall’Osto et al. 2017). Thus, the efficiency of monomeric LHCs in inducing thylakoid stacking was higher than that of trimeric LHCII. Similar conclusions can be drawn by comparing *koLHCII* and *lowLHCII,* which have the same LHC/PSII core stoichiometry (Guardini et al. 2025), LHC monomers are at least 3 times more efficient at stabilizing grana than LHC trimers.

Pigment circular dichroism (CD) spectroscopy, a sensitive and nondestructive method for probing thylakoid membranes architecture (Lambrev and Akhtar 2019), confirmed that selective loss of LHC subunits led to significant changes in membrane macro-organization and a marked reduction in thylakoids stacking (see Figure S7 for details).

To investigate the stacking capacity of individual monomeric Lhcb, we took advantage of the *ch1* and *ch1 koLhcb5* genotypes, which lack CAO (Chl *a* oxygenase) and have a significantly reduced antenna size (Kim et al. 2009) caused by the destabilization of most Lhcb proteins in the absence of Chl *b,* the only exception being Lhcb5 (Croce et al. 2002). The stacking in the Chl b-less *ch1* mutant (Fig. 2f) was only slightly lower compared to the *koLHCII* genotype (Fig. 2k), implying that the factors stabilizing grana in *koLHCII* were also present in *ch1.* In contrast, the grana cross-sectional area was drastically reduced in the *ch1 koLhcb5* genotype (Fig. 2g, k), indicating that Lhcb5 had a prominent function in grana stabilization relative to LHCII. Furthermore, the chloroplast area and the diameter of grana sections were similar in both *ch1* and *ch1 koLhcb5* genotypes (Fig. 2h, i).

**Figure 3:**
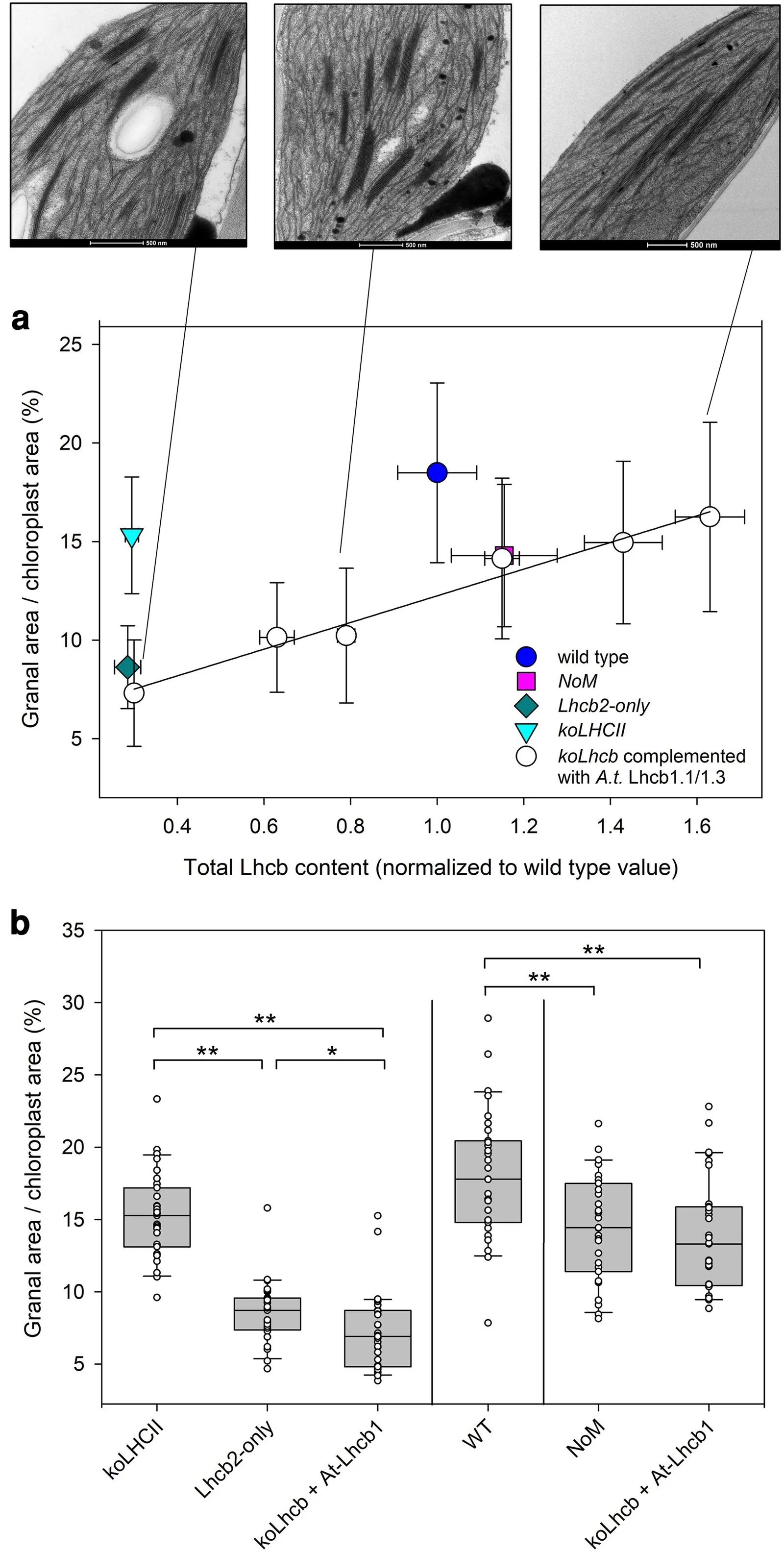
Correlation between granal area *per* chloroplast area and LHC content in plants expressing different types and amounts of Lhcb proteins. Plants grown under short-day conditions were dark-adapted for 2 h before harvesting their leaves. Samples were then fixed, embedded, and examined in thin sections at various magnifications. For each genotype, Lhcb content was quantified by SDS–PAGE followed by Coomassie staining and densitometric analysis. **(**a**)** The extent of thylakoid stacking was statistically evaluated by measuring the granal area relative to the chloroplast area, measured from individual micrographs (*n* = 30). For correlation analysis, data points from *A. thaliana koLhcb* complemented with WT Lhcb1.1/1.3 (black circles) were fitted with a linear function (black line). The genotypes *koLHCII*, *NoM,* and *Lhcb2-only* were included as references. Error bars represent standard deviation (SD). **(**b**)** Comparison of grana cross-sections among lines with matched Lhcb content. Box plots are grouped into panels containing genotypes with equivalent total Lhcb content, corresponding to ∼30%, 100%, and ∼120% of wild-type (WT) levels. All individual data points are shown; in the box plots, the boxes indicate the interquartile areas, the horizontal lines show the medians, the whiskers are drawn down to the 5th percentile and up to the 95th. The statistically significant mean differences (*t*-test) are marked by asterisks indicating the following confidence intervals: *≤95%; **≤99.9%. Exact *P*-values are provided in the source data file.

All these results highlight the distinct roles of different PSII antenna system components in promoting thylakoid stacking. To assess the contribution of each, we compared the grana cross-section among genotypes with similar overall Lhcb content. We analyzed *Arabidopsis* lines obtained by complementing *koLhcb* plants with *A.t. Lhcb1.1/1.3* sequences and ranked them based on their Lhcb content, which ranged from 30% to 170% of the wild type level. Figure 3 shows that the ratio of granal *vs*. chloroplast area increased steadily with Lhcb1 accumulation. When comparing the effect of LHC accumulation on grana cross-section, both wild type and *koLHCII* lines exhibited significantly higher grana content than plants expressing Lhcb1 at comparable LHC levels, confirming that monomeric antennae provide higher stacking efficiency. The genome editing of *Lhcb1* in the *NoM koLhcb3* background (Figure S1) produced the *Lhcb2-only* genotype, in which Lhcb2 is the sole PSII antenna (Ordon et al. 2020). Interestingly, the efficiency of Lhcb2 in grana stabilization was significantly higher than that of Lhcb1 (Fig. 3).

### Phosphorylation of Lhcb2 affects grana stacking during state transitions

**Figure 4:**
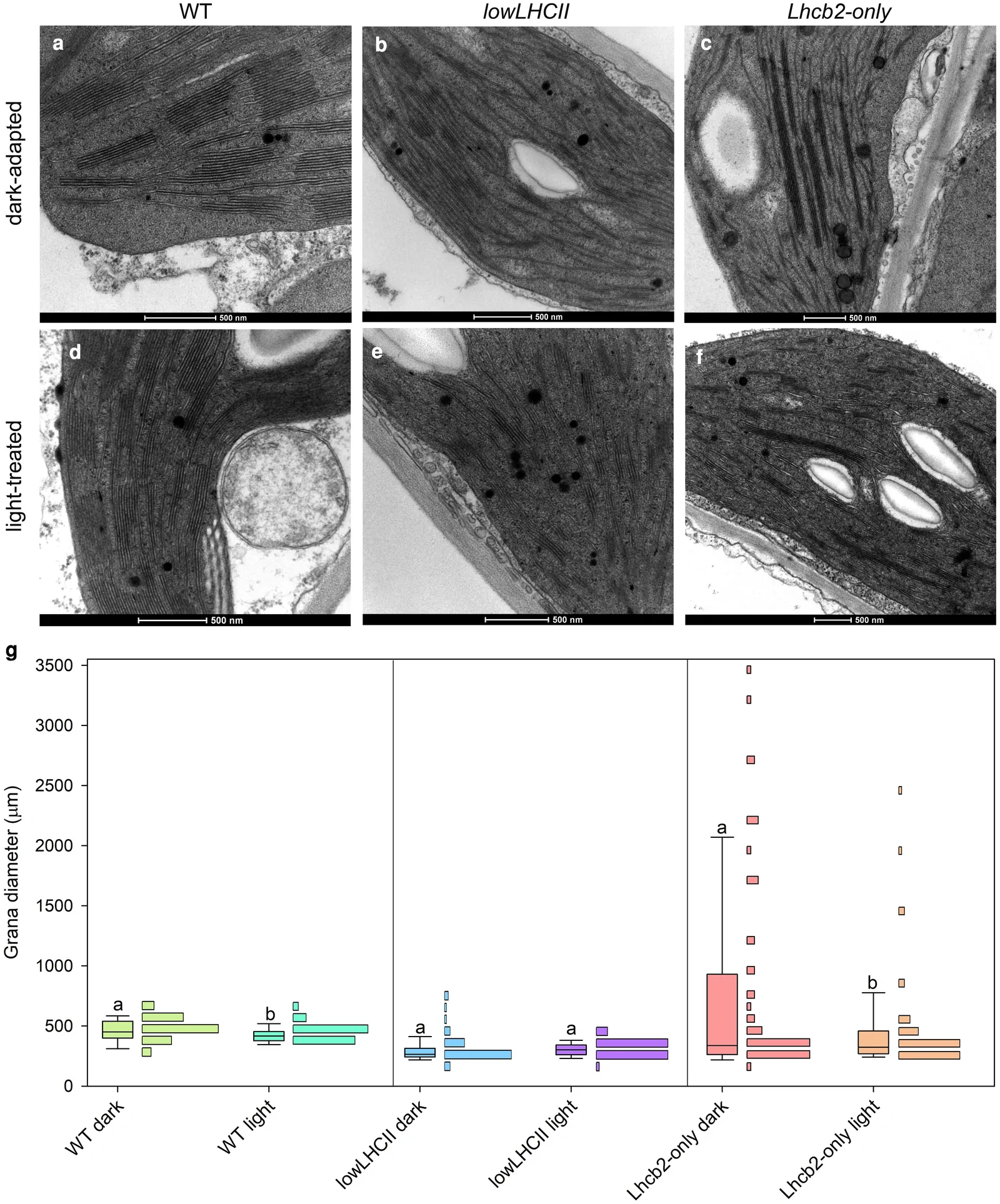
Changes in chloroplast ultrastructure of state 1− and state 2-adapted leaves. (a–f**)** Leaves from wild type (a, d), *lowLHCII* (b, e) and *Lhcb2-only* (c, f) plants were subjected to different treatments. Panels a–c show leaves that were dark-adapted for 2 h, while panels d–f depict leaves exposed to 60 min of PSII light (200 µmol photons m^−2^ s^−1^), promoting LHCII phosphorylation and the transition to state 2. Leaf discs were then fixed, embedded, and observed in thin sections. **(**g**)** Statistical analysis of differences in grana diameter in state 1 and state 2, measured from individual grana (*n* = 55 [wild type], 58 [*lowLHCII*], 60 [*Lhcb2-only*]). Histograms show the frequency distributions of the numbers of thylakoids per granum in the different genotypes/conditions. In the box plots, the boxes indicate the interquartile range, the horizontal lines show the medians, and the whiskers extend to the 5th and 95th percentiles. Values that differ significantly (ANOVA followed by Tukey's post-hoc test at a significance level of *P* < 0.05) are indicated with different letters. All experiments were independently repeated twice, yielding similar results.

Lhcb2 plays a key role in mediating state 1—state 2 transitions (Cutolo et al. 2023). We analyzed grana membrane organization in the *Lhcb2-only* line (Fig. 4a–f) and included the genotype *lowLHCII* as control with comparable Lhcb content (Figure S1). In dark-adapted samples, wild type and *lowLHCII* chloroplasts exhibited grana diameters ranging from 300 to 650 nm (Fig. 4g) (Kirchhoff 2019). Despite similar PSII-core/LHCII ratios, dark-adapted *Lhcb2-*only plants differed markedly from *lowLHCII* lines in that they displayed much larger grana, with diameters up to 3 µm or more (Fig. 4a–c, g), spanning entire chloroplast cross-sections. To investigate the effect of light on thylakoid stacking, dark-adapted leaves were exposed to PSII light (200 µmol photons m^−2^ s^−1^, Fig. 4d–f) (Pfannschmidt et al. 1999), promoting PQ over-reduction in both *Lhcb2-only* and *lowLHCII* genotypes, and leading to Lhcb2 phosphorylation (Figure S8). In *Lhcb2-only* chloroplasts, the oversized grana largely disassembled upon illumination, and the size distribution shifted toward smaller diameters. Induction of Lhcb2 phosphorylation significantly reduced both grana diameter and height in both the *Lhcb2-only* and *lowLHCII* lines (Fig. 4g, Figure S9). This result points to Lhcb2 as a major source of dynamics in thylakoid membranes. It is tempting to attribute such behavior to the Lhcb2 property of undergoing reversible phosphorylation and thus change the surface charge distribution. However, Lhcb1 also undergoes phosphorylation without causing such strong thylakoid organization change (Cutolo et al. 2023), highlighting a specific effect of Lhcb2 sequence that deserves future analysis.

### Physiological consequences of Lhcb depletion for photosynthesis

LHCs have been proposed as key determinants for several regulatory responses that are critical in the challenging terrestrial light environment, including the PSII repair cycle, the balance between CEF and LEF, and mechanisms that maintain an appropriate distribution of excitation energy between the PSs (González et al. 2021). We therefore assessed whether Lhcb depletion affected the PSII repair rate. Measurements of PSII quantum yield after photoinhibitory treatment (see Figure S10a) indicated that both wild type and mutant leaves retained an active PSII repair mechanism, although PSII activity in the *koLhcb* recovered more slowly.

Figure S10b shows the relationship between the total amplitude of the electrochromic shift (ECS_t_), which reflects the light-driven protonmotive force (pmf) across the thylakoids, and the fraction of pmf generated by LEF alone (pmfLEF) in leaves. The slope of the linear relationship between pmfLEF and ECS_t_ is proportional to the electron-to-proton transfer stoichiometry (Avenson et al. 2005). Our results indicate that wild type and mutant plants generated a similar level of pmf attributable to LEF alone, consistent with comparable CEF contributions in all genotypes.

**Figure 5:**
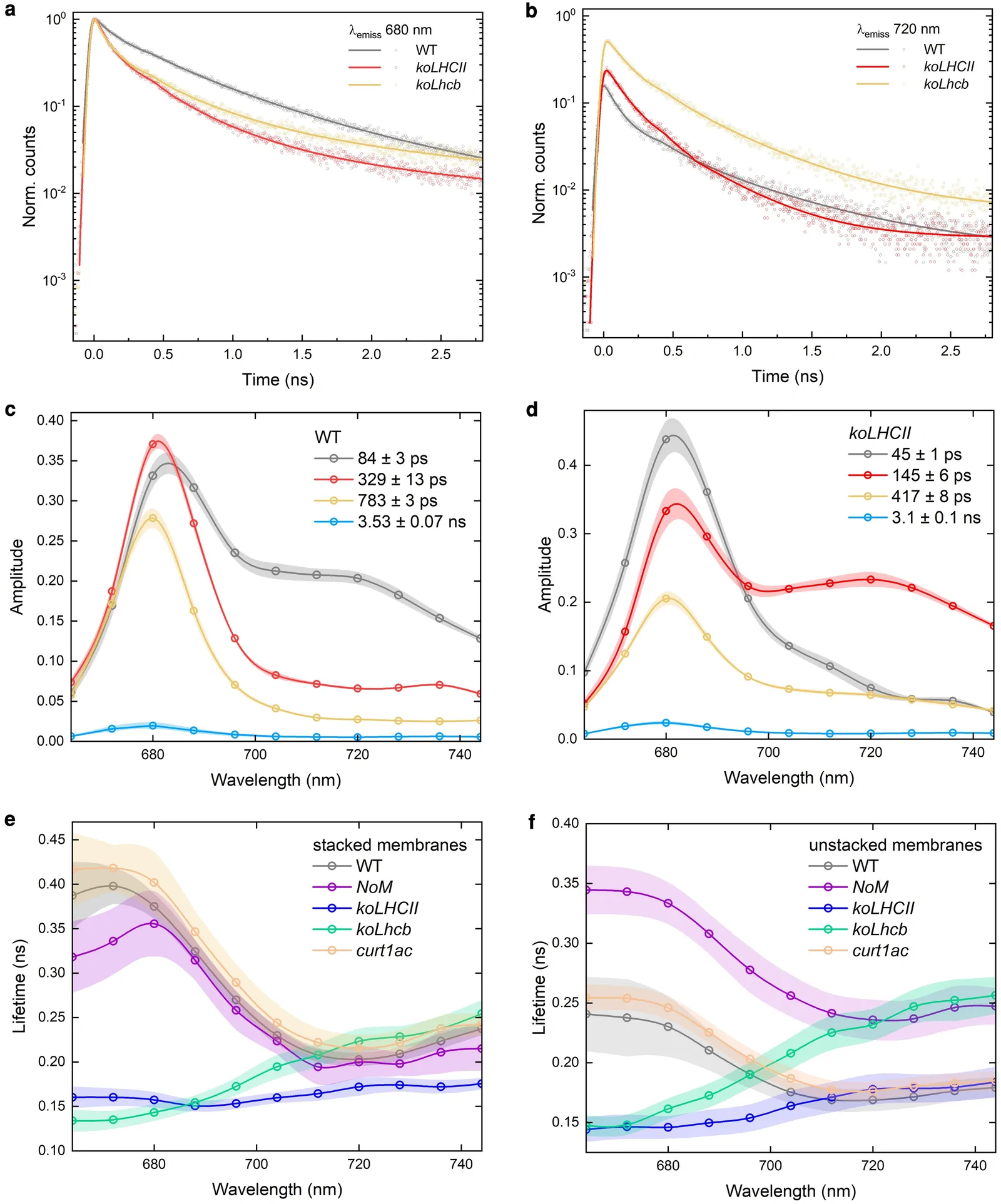
Evaluation of spillover in thylakoid membranes by time-resolved fluorescence. (a–b) Fluorescence decay kinetics recorded at 680 nm (a) and 720 nm (b) from thylakoid membranes of WT, *koLHCII* and *koLhcb* plants resuspended in stacking buffer medium. The decay profiles are normalized to the maximum fluorescence at 680 nm. Data points represent the measured values, while the lines indicate multiexponential fits. (c–d) Decay-associated spectra (DAS) of the fluorescence kinetics for WT (a) and *koLHCII* (B). The shaded areas in the spectra represent standard errors (*n* = 3 to 4). (e–f) Wavelength dependence of the amplitude-weighted average fluorescence lifetime $\tau_{av}=\underset{i}{\sum }\;a_{i}\tau_{i}/\underset{i}{\sum }\;a_{i}$ for the stacked (e) and unstacked (f) membranes. The longest-living, ns-lifetime component assigned to free pigments/LHCII was excluded from the calculation of average lifetimes.

To assess the impact of different LHC deletions on excitation energy spillover from PSII to PSI, we performed time-resolved fluorescence spectroscopy on thylakoid membranes isolated from wild type, *NoM*, *koLHCII*, *koLhcb*, and the *curt1ac* mutant. In *curt1ac*, thylakoids are disorganized, with extended regions of unstacked membranes and broader stacks containing fewer layers than in the wild type (Armbruster et al. 2013) while the major thylakoid multiprotein complexes accumulated to wild type levels (Figure S11). Time-resolved spectroscopy enables PSI and PSII emissions to be distinguished based on differences in their spectra and decay lifetimes (Bag et al. 2020; Akhtar et al. 2024): PSII emits mainly around 680 nm, whereas PSI shows a broad emission band around 720 nm (Slavov et al. 2008). When excited independently, PSII fluorescence at 680 nm displays long decay lifetimes ranging from ∼300 ps to 2 ns (Miloslavina et al. 2006), whereas PSI emission at 720 nm decays much faster, with 60 to 80 ps lifetimes (Wientjes et al. 2013; Akhtar et al. 2018). Under spillover conditions, PSII fluorescence at 680 nm is expected to decay more rapidly, accompanied by increased emission at 720 nm (Akhtar et al. 2024; Kim et al. 2023). Both effects were observed in *koLHCII* and *koLhcb* relative to the wild type (Fig. 5a, b, Figure S12): PSII fluorescence decay at 680 nm was markedly accelerated, and the 720 nm emission increased, particularly in *koLhcb*.

Global lifetime analysis of the fluorescence decay kinetics in the 664 to 744 nm range yielded 4 decay components and their corresponding decay-associated emission spectra (DAES). In wild-type thylakoids, the fastest component (84 ps) exhibited a broad shoulder around 720 nm, attributed to PSI-LHCI, whereas the longer-lived components (330 and 780 ps) were assigned to PSII-LHCII, indicating efficient lateral segregation and energetic uncoupling of the 2 PSs (Fig. 5c). Resuspending of thylakoids in a Mg^2+^-free buffer induces membrane unstacking (Kiss et al. 2008), disrupts lateral segregation and enhances spillover. Under these conditions, PSII decay at 680 nm accelerated, whereas PSI emission at 720 nm decayed more slowly (Figure S12), consistent with observations in spinach thylakoids (van der Weij-de Wit et al. 2007).

Spillover was also evident from the wavelength dependence of the average fluorescence lifetime. With segregated PSs, as in wild-type thylakoids, the lifetime in the 680 to 690 nm range is substantially longer than in the far-red region, because the former is dominated by PSII and the latter by PSI. When spillover occurs, PSII lifetimes shorten, and this wavelength dependence becomes less pronounced. This pattern was also observed upon resuspension in unstacking medium (Figs 5e, f).

Marked changes were observed in the DAES of the antenna-depleted genotypes (Fig. 5, Figure S12). In *koLHCII*, the shortest-lived component had a lifetime of 45 ps and a DAES lacking a distinct LHCI emission band in the far-red region; instead, the red Chls emission decayed with a lifetime of 145 ps, indicating more extensive spillover than in the wild type (Fig. 5d). These changes can be interpreted using a kinetic model of spillover (Figure S13), in which PSI is represented by 2 kinetic compartments (bulk and red Chls), and PSII by three (antenna excited states and 2 charge-separated states). In the absence of spillover, PSI decays with lifetimes of 17 and 78 ps, with main PSII decay components at approximately 300 and 600 ps. If a small fraction (20 to 30%) of PSs are energetically coupled, the resulting DAES qualitatively resemble those of unstacked wild type thylakoids. By contrast, the DAES of *koLHCII* thylakoids are better reproduced when a larger fraction of PSII and PSI are energetically connected (Figure S14).

The *koLhcb* mutant showed large differences in both DAES and average lifetimes compared with the wild type. The 120 and 420 ps components peaked at 720 nm, indicating a reduced contribution of PSII to the overall fluorescence. Nonetheless, the relatively long-lived far-red emission can be explained by energetic equilibration between PSI and PSII, supported by the wavelength dependence of the average lifetime and by the insensitivity of the DAES to the resuspension medium (Figure S12). *NoM* and *curt1ac* mutants behaved similarly to wild type, suggesting that lateral segregation remains mostly intact despite differences in thylakoid macro-organization. Notably, *NoM* membrane fluorescence was highly sensitive to unstacking. Under stacking conditions, the 680 nm decay included a ∼50% contribution from free LHCII, with a lifetime of 1.9 ns (Figure S12), comparable to LHCII fluorescence in lincomycin-treated plants (Belgio et al. 2012). Upon resuspension in unstacking buffer, this long-lived component disappeared and was replaced by a 300 ps decay component with a maximum at the PSI wavelength. This indicates that, in the absence of Mg^2+^, LHCII domains lose their stacking capacity, enabling PSI to effectively quench excitations in the free LHCII pool (Wientjes et al. 2013; Akhtar et al. 2016). When the ns component from free LHCII was excluded, the average fluorescence lifetime showed the same wavelength dependence as in the wild type.

In summary, time-resolved fluorescence spectroscopy revealed pronounced excitation energy spillover in the LHC-depleted genotypes *koLHCII* and, particularly, *koLhcb*. The pigment compositions of PSI-LHCI and PSII core complex were similar in all genotypes (Table S2), ruling out the possibility that differences in excitation energy transfer dynamics between PSII and PSI arise from altered pigment composition of the supercomplexes.

By contrast, *NoM* and *curt1ac*, despite exhibiting substantial alterations in thylakoid organization, retained energetic separation between the PSs, or displayed spillover levels comparable to the wild type, provided that Mg^2+^ was present to support membrane stacking.

## Discussion

### Thylakoid membranes stacking

In this study, knockout of LHC genes led to thylakoid de-stacking and reduced lateral heterogeneity between PSI and PSII. EM analysis showed that deletion of both PSII antenna types resulted in agranal chloroplasts (Fig. 2d) while deletion of either monomeric or trimeric LHCII yielded a reduction of membrane stacking by 40% respect to the value observed in *koLHCII*. Since LHCII trimers are far more abundant than monomeric LHCs, this suggests the latter are far more effective at promoting membrane stacking (Figure S15). This conclusion is further supported by analysis of a population expressing increasing levels of Lhcb1 trimeric complexes (Fig. 3).

Analysis of Chl *b*-less genotypes further pinpointed the antenna components involved in membrane stacking. In the *ch1* mutant, in which the absence of Chl *b* destabilizes most LHC complexes, the grana-to-chloroplast surface ratio was similar to that of *koLHCII* (Fig. 2k). Notably, Lhcb5 remains present and functional in *ch1* plants (Kim et al. 2009) as it can bind Chl *a* at most Chl *b* sites (Croce et al. 2002). The *ch1 koLhcb5* double mutant exhibited a strong reduction in grana abundance and cross-sectional area (Fig. 2j, k), suggesting that Lhcb5 is a critical determinant of grana formation. Moreover, the stacking phenotype of *Lhcb2-only* plants demonstrated that Lhcb2 is crucial for modulating membrane appression: induction of Lhcb2 phosphorylation by PSII light treatment (Fig. 4d–f, Figure S8) significantly reduced both diameter and height (Fig. 4, Figure S9). Notably, ko lines lacking either Lhcb2 or Lhcb5 alone did not display major changes in thylakoid structure (Figure S16), suggesting that the missing complexes are functionally compensated by the remaining Lhcb subunits, which saturates the extent of stacking, which is likely to be limited by the relative abundance of PSII (Belgio et al. 2015).

Structural studies of purified dimeric PSII supercomplexes interacting via their stromal sides (Albanese et al. 2020) suggested that both monomeric and trimeric LHCs contribute to stabilizing interactions between PSII supercomplexes facing each other across thylakoid partitions. However, our in vivo data identified Lhcb5 and Lhcb2 rather than Lhcb1 and Lhcb4 as the main contributors to grana stabilization and dynamic regulation. This discrepancy likely reflects the use of isolated dimeric PSII supercomplexes sides (Albanese et al. 2020), which model face-to-face PSII interactions but does not integrate the contributions of lateral interactions within the membrane plane which are also required to stabilize native PSII organization within grana. Moreover, alternative face-to-face dimer arrangements have been reported (Grinzato et al. 2020). Other high-resolution studies revealed distinct conformations of stacked PSII C2S2-C2S2 megacomplexes stabilized through interactions between Lhcb5 and an LHCII subunit (Caspy et al. 2023). The 10 kDa PSII core subunit PsbR has also been implicated in maintaining PSII-LHCII megacomplexes integrity through interactions with D1, D2, and CP43 (Shan et al. 2024), and was proposed to promote stacking of adjacent grana thylakoids by mediating vertical interactions (Albanese et al. 2017; Shan et al. 2024). Consistently, *Arabidopsis* mutants lacking PsbR show perturbed energy transfer dynamics between LHCII and the PSII core (Niedzwiedzki et al. 2025). Intriguingly, we observed a marked reduction in PsbR abundance in *koLhcb* and *NoM* thylakoids, whereas PsbR levels were unchanged in *koLHCII* and *curt1ac* mutants (Figure S17), despite their altered grana morphology (Fig. 2, Figures S11). These findings suggest that weakened association of PsbR with the PSII core in the absence of monomeric Lhcb may underlie the higher stacking capacity of monomeric LHC compared with trimers (Figs 2 and 3, Figure S15).

### Functional significance of granal stacking

Grana stacking is thought to be fundamental for an efficient PSII repair, transition between LET and Cyclic Electron Transport CET and lateral segregation of PSII and PSI, thus preventing spillover (Trissl and Wilhelm 1993). Our physiological analysis showed that PSII repair was slower in *koLhcb* than in *koLHCII* (Figure S10a). This difference could arise from either the absence of monomeric LHCs or the lack of grana; we favor the latter, as the *NoM* genotype displays recovery kinetics similar to the wild type (Dall’Osto et al. 2020). No major differences in the CET/LET ratio were detected among the genotypes (Figure S10b). Finally, we consider whether excitonic spillover from PSII to PSI occurs upon thylakoid de-stacking. Time-resolved fluorescence measurements showed that the presence of Lhcb complexes prevents PSII-to-PSI spillover, likely by preserving lateral segregation and shielding PSII from direct interaction with PSI. The long-lived decay components (120 ps and 420 ps) in the far-red spectral region of LHC-depleted membranes can be assigned to PSI complexes receiving excitation from PSII (Figure S14). Excitation energy spillover was pronounced in *koLHCII* and even stronger in *koLhcb*. This is expected to severely disturb the excitation balance between the 2 PSs and thereby impair linear electron flow from H_2_O to NADPH, consistent with the observed retarded growth phenotype (Fig. 1, Figure S5).

Interestingly, both *NoM* and *curt1ac* mutants showed low levels of spillover despite their strongly altered thylakoid architectures (Fig. 5e, Figure S11). The *curt1ac* phenotype is particularly informative: the presence of “pseudo-grana”, ie thylakoid stacks that still accommodate PSII supercomplexes, appears sufficient to sustain PSII function (Armbruster et al. 2013), similar to green microalgae, which lack well-defined grana margins (Wietrzynski et al. 2020). Also, previous studies showed that *curt* mutants display delayed growth under fluctuating or stressful light conditions (Pribil et al. 2018) in close agreement with our results (Figure S11d, e).

It could be argued that spillover in *koLhcb* is a mere artifact of the altered thylakoid architecture, where the absence of grana (Fig. 2d) allows PSI and PSII to co-localize. Alternatively, spillover could serve a physiological role in wild-type chloroplasts—helping to balance excitation between the 2 PSs in concert with state transitions (Allen and Forsberg 2001). Excitation energy transfer from PSII to PSI has been reported in cyanobacteria (Akhtar et al. 2024) and in red algae (Ueno et al. 2016), where it helps maintain balanced energy flow between PSs. Spillover has also been reported to varying extents, in PSI–PSII megacomplexes of higher plants, particularly under EL conditions (Yokono et al. 2015). Under low light, chloroplasts maximize light capture by forming large, tightly stacked grana, whereas under EL they adopt fewer and smaller stacks and reduce antenna content (Anderson et al. 2012). EL-induced acclimation may well modulate the level of energy spillover in vivo. Under such conditions, transferring a fraction of excitation energy between PSs could act as a safety valve to relieve excitation pressure on PSII, as proposed for control of photoinhibition in lichens (Slavov et al. 2013) and in the evergreen conifer *Pinus sylvestris* where extensive thylakoid de-stacking has been proposed to promote direct PSII-PSI interactions by mixing thylakoid domains, enabling “sustained quenching” mechanism that protects evergreens in winter (Bag et al. 2020). Future work is needed in order to assess the contribution of spillover relative to other photoprotective mechanisms in land plants depending on growth conditions.

### Driving forces mediating grana stacking

Our results show that Lhcb complexes are essential to stabilize grana stacking. However, the precise mechanisms underlying this process remain to be fully clarified. Multiple lines of evidence indicate that grana formation is a self-organizing process, where the physicochemical interactions among thylakoid membrane proteins and lipids are sufficient to account for membrane stacking, without the need for dedicated assembly factors (Staehelin 1976; Kirchhoff et al. 2007). In this view, stacking can be explained by the force balance between van der Waals attraction and screened electrostatic and steric-hydration repulsion (the “Delocalized forces model”) (Krysiak et al. 2026). Alternatively, specific protein–protein interactions between the stroma-facing surfaces of PSII-LHCII complexes have been proposed to stabilize membrane architecture (Albanese et al. 2020). However, this hypothesis is challenged by the observation that the PSII–LHCII complexes in adjacent appressed membranes are randomly distributed with minimal overlap (Wietrzynski et al. 2025), and that the partition distance is significantly larger than observed in isolated PSII–LHCII dimers (Li et al. 2020).

To conclude, we propose that the stroma-exposed domains of Lhcb subunits, particularly the monomeric ones, play a decisive role in modulating this attraction-repulsion balance. In this view, membrane stacking is governed by a balance of long-range, diffuse physicochemical forces that are established and tuned by stroma-exposed domains of specific Lhcb proteins. Future work will involving site specific mutagenesis of charged residues within N-terminal domains will be required to identify critical factors driving grana stacking.

## Materials and methods

### Plant materials

*Arabidopsis thaliana* mutants *ch1*, *ch1 koLhcb5*, *koLhcb3,* and *NoM* were obtained as previously described (Havaux et al. 2007; Damkjaer et al. 2009; Dall’Osto et al. 2014)*. NoM koLhcb3* was obtained by crossing single mutants and selecting progeny by immunoblotting. *koLHCII*, *Lhcb2-only*, *koLhcb,* and *lowLHCII* lines were obtained by genome editing as reported in (Guardini et al. 2025). Genome-edited seedlings were tested for resistance to hygromycin (25 mg l^−1^). For each genotype, independent transformants (T1 generation) were self-fertilized, and the absences of proteins were confirmed in the T3 generation by immunotitration. Lines expressing differential amounts of Lhcb1 were obtained by complementing the *koLhcb #16* plants with the *pK7WG2-LHCB1* plant destination vector, carrying the genomic sequence encoding Lhcb1.1 and Lhcb1.3, along with their respective regulatory elements. The genomic fragment (4,913 bp) was amplified by PCR (EURx Hybrid DNA polymerase) with the primer pair: *PmeI-Lhcb1-FW:* AGCTTTGTTTAAACTCGATACCACACACCAATGACAC and *KpnI-Lhcb1-RV:* GGGGTACCACAAATGTGTTTGATTTGTACGGAT and cloned with PmeI and KpnI restriction enzymes (*New England Biolabs*) into the pK7WG2 vector. Transformed seeds were selected on kanamycin (50 mg l^−1^). Several independent T1 transformants were self-fertilized, and Lhcb1 abundance was assessed in the T2 generation by SDS-PAGE and Coomassie-staining.

### Growth conditions

Wild type and mutant genotypes were grown in soil, in a phytotron for 5 wk at either 120 or 400 μmol photons m^−2^ s^−1^, 23 °C, 70% relative humidity, 8/16 h of day/night. All biochemical and physiological analyses were performed on plants before the onset of flowering. The growth of plants was determined by measuring the rosette area twice a week with ImageJ software (Schneider et al. 2012) and the rosette fresh weight at the end of the growth cycle.

### Membrane isolation

Thylakoid membranes were isolated as previously described (Casazza et al. 2001). Measurements were performed on thylakoid membranes in both stacked and unstacked states. Stacked thylakoid membranes were prepared by resuspension of freshly isolated thylakoid membranes in a buffer containing 0.3 M sorbitol, 2.5 mM EDTA, 5 mM MgCl_2_, 10 mM NaHCO_3_, 20 mM HEPES (pH 7.6), and 0.5% (w/v) fatty acid-free BSA to an optical density of 0.5 at red maximum. To obtain unstacked thylakoid membranes, the same amount of sample was resuspended in a buffer containing 20 mM HEPES (pH 7.6).

### Pigment analysis

Pigments were extracted from leaf discs or purified complexes with 85% acetone buffered with Na_2_CO_3_, then separated and quantified by HPLC (Jasco Extrema LC-4000) as in (Gilmore and Yamamoto 1991).

### Absorption and circular dichroism spectroscopy

Absorption spectra were recorded at RT using an Aminco DW-2000 spectrophotometer. CD spectra in the range of 350 to 750 nm were recorded with a J-815 spectropolarimeter (Jasco, Japan). The measurements were performed in a quartz cuvette of 1 cm optical path length with 2 nm spectral bandwidth, an integration time of 2 s and scanning speed of 100 nm min^−1^.

### Time-resolved fluorescence (TRF) spectroscopy

Picosecond time-resolved fluorescence measurements were performed using a time-correlated single-photon counting setup (FluoTime 200/PicoHarp 300 spectrometer, PicoQuant) equipped with a microchannel plate detector (R3809U, Hamamatsu, Japan). Excitation pulses were provided by a Fianium WhiteLase Micro supercontinuum laser (NKT Photonics, UK) operating at a repetition rate of 20 MHz. Excitation at 440 nm was selected using a monochromator and a band pass filter. Fluorescence emission was detected through a second monochromator over the 664 to 744 nm range, in 8 nm steps, using 1 mm excitation and detection slit widths. All the samples (stacked and unstacked membranes) were diluted to an absorbance of 0.04 at the excitation wavelength and continuously circulated through a 1 mm pathlength flow cell at a flow rate of 4 mL/min to avoid repeated excitation of the RC. The buffer of the thylakoid membranes was supplemented with 25 µM dichlophenolindophenol and 0.5 mM potassium ferricyanide. Alternatively, measurements with closed PSII RCs were performed by adding 20 µM DCMU to the medium and reducing the flow rate ∼10-fold.

The instrument response function (IRF), measured using 1% Ludox as a scattering solution, had a full width at half maximum of 40 ps. All measurements were carried out at room temperature (RT, 24 °C). The data were corrected for the detector's spectral response using a calibrated light source (ESC-842, Jasco) as a reference. Global multiexponential lifetime analysis with IRF deconvolution was performed using custom MATLAB routines. Decay lifetimes and wavelength-dependent IRF time shifts were determined by nonlinear least-squares minimization of the squared sum of the Poisson-weighted residuals, and the pre-exponential amplitudes were obtained by linear least-squares fitting (van Stokkum et al. 2004).

### Electrophoresis and immunoblotting

SDS-PAGE analysis of thylakoid proteins was performed using the Tris-Tricine buffer system (Schägger and von Jagow 1987). For immunotitration (Towbin et al. 1979), proteins were detected with alkaline phosphatase-conjugated antibody (Sigma-Aldrich A3687). Primary antibodies used were: α-PsbB/CP47 (AS04 038), α-Lhcb1 (AS01 004), α-Lhcb2 (AS01 003), α-P-Lhcb1 (AS13 2704), α-P-Lhcb2 (AS13 2705) from Agrisera. Signal amplitude was quantified using the GelPro 3.2 software. Non-denaturing Deriphat-PAGE was performed as in (Havaux et al. 2007), using an acrylamide linear gradient from 4% to 12%.

### In vivo spectroscopic assays

PSII function during photosynthesis was measured through Chl fluorescence (Baker 2008) on whole leaves at RT with a DUAL-PAM-100 equipment (Walz, GmbH). Steady state, light-induced pmf was estimated from changes in absorbance associated with the ECS at 520 nm (Livingston et al. 2010), using a LED spectrophotometer (JTS10; Biologic Science Instruments). PSII repair efficiency after photooxidative stress was measured as in (de Bianchi et al. 2011). PSII operating efficiency (ϕ_PSII_) and the fraction of open PSII centers (qL) were calculated as described by (Baker 2008) at RT with a HEXAGON-IMAGING-PAM (Walz, GmbH).

### Electron microscopy and image analysis

Transmission electron microscopy on leaf fragments was conducted using a FEI Tecnai T12 electron microscope operating at 100 kV accelerating voltage. Leaf fragments were fixed in 3% glutaraldehyde in 0.1 M cacodylate buffer pH 6.9. For each genotype, tip regions of 4 independent fully developed leaves were sampled (Gügel and Soll 2017) and dark-adapted for 2 h. Grana were defined as made of minimum 3 thylakoids stacks (Gügel and Soll 2017). Grana morphological parameters were obtained from micrographs using ImageJ, upon manual measurements (Schneider et al. 2012). Grana diameter (d) was determined as the distance between left and right end of the granum, in the case of irregular grana variable diameters were considered and averaged; grana height (h) was determined as the distance between the top and bottom layers of the granum end membrane, in the case of irregular grana variable diameters were considered and averaged, following the methodologies outlined in (Ioannidis et al. 2009; Mazur et al. 2021; Colpo et al. 2023) a schematic representation is reported in Figure S18a. The granal area/chloroplast area ratio (Kim et al. 2009) was calculated as reported in Figure S18b. The number of grana per chloroplast was measured using the multiple-point function of ImageJ (Figure S18c). Sample size and kind of replicate is described in the legend of each figure.

### LHCII phosphorylation induction

Leaves were dark adapted for 60 min prior to exposure to 200 μmol photons m^−2^ s^−1^ PSII light for 60 min at 24 °C. PSII light was provided by a cool-white lamp equipped with an orange-yellow filter (Lee filters, 105 Orange) as described by (Pfannschmidt et al. 1999).

### Sucrose gradient fractionation

Sucrose gradient ultracentrifugation was conducted as reported by (Capaldi et al. 2025). Gradients were formed directly in the tubes upon freezing (−80 °C) and thawing (+4 °C) a 0.5 M sucrose solution supplemented with 10 mM HEPES pH 7.5 and 0.03% dodecyl-β-d-maltoside (β-DM).

### Statistics

Statistical analyses were performed in SigmaPlot using *t*-test or 1-way ANOVA, means were separated with Tukey's post hoc test at a significant level of *P* < 0.05 (see the figure legends for details).

### Accession numbers

Sequence data from this article can be found in the *Arabidopsis* Genome Initiative or GenBank/EMBL databases under accession numbers At1g29920 (*Lhcb1.1*), At1g29910 (*Lhcb1.2*), At1g29930 (*Lhcb1.3*), At2g34430 (*Lhcb1.4*), At2g34420 (*Lhcb1.5*), At2g05100 (*Lhcb2.1*), At2g05070 (*Lhcb2.2*), At3g27690 (*Lhcb2.3*), At5g54270 (*Lhcb3*), At5g01530 (*Lhcb4.1*), At3g08940 (*Lhcb4.2*), At4g10340 (*Lhcb5*), At1g44446 (*cao*). The KO lines used in the work were obtained from the NASC under the stock numbers N376476 (*koLhcb4.1*), N877954 (*koLhcb4.2*), N514869 (*koLhcb5*), N520342 (*koLhcb3*), N524295 (*ch1*).

## Supplementary Material

koag256_Supplementary_Data

## Data Availability

The data underlying this article are available in the article and in its online supplementary material.
