# Supplementary material for: Specific light-harvesting complexes mediate grana stacking and prevent energy spillover between photosystems in plant chloroplasts
Source: Plant Cell. 2026 Sep 1;38(9):koag256. doi: 10.1093/plcell/koag256 (PMC13564224; doi:10.1093/plcell/koag256)
Supplement: Supplementary file 1 [file koag256_supplementary_data.zip › SI_Guardini_stacking.docx]

**Supplemental Information**

**Supplementary Figure S1**

**Supplementary Figure S1. Schematic diagram of protein composition of *Arabidopsis* LHC mutants developed in this study.** (A) The *NoM* line was initially obtained as reported (Dall’Osto et al. 2017), with an additional T-DNA insertion in At5g54270 (Lhcb3; NASC N520342) further introgressed. To delete *Lhcb1* or *Lhcb2* genes, CRISPR-Cas9 targeted mutagenesis strategy was implemented according to (Ordon et al. 2020), in both (I) *koLhcb3* and (II) *NoM koLhcb3* genetic backgrounds: for each of the 5 *Lhcb1* and 3 *Lhcb2* genes, pairs of sg (single guide) RNAs were designed within coding sequences (Supplementary Figure S2, Table S1), with the aim of producing large DNA deletions, favoring DNA repair by non-homologous end-joining (NHEJ) (Malzahn et al. 2017). *Lhcb1* and *Lhcb2* genes were separately targeted to get Lhcb1-less and Lhcb2-less plants, which were then crossed to obtain lines lacking both Lhcb1 and Lhcb2 proteins, followed by progeny selection through immunoblotting. We obtained either (I) the *koLHCII* line (lacking all LHCII but retaining monomeric Lhcb4-6 proteins), or (II) the *koLhcb* line, lacking both the monomeric and trimeric LHCs of PSII, by applying this procedure on *koLhcb3* or *NoM koLhcb3* genotypes. In both cases LHCI proteins were unaffected. *Lhcb2-only* lines were obtained by deleting *Lhcb1* genes in *NoM koLhcb3* plants (III). *low-LHCII* lines were developed by crossing *NoM koLhcb3* lines that underwent limited genome editing at the *Lhcb1* and *Lhcb2* loci, selecting progeny that retained about 30% of the LHC content of the parent genotype (IV). (B) SDS–PAGE analysis of wild type (WT) and LHC mutant thylakoid proteins, performed using the Tris-Tricine buffer system. Selected apoprotein bands are marked. (C) Lhcb content in wild type and mutant leaves. Monomeric Lhcb were quantified by SDS-PAGE and normalized to the PSII monomeric core complex (data adapted from (Guardini et al. 2025)). The amount of LHCII trimers was estimated from the PSI/PSII ratio (Guardini et al. 2025), the Chl a/b ratios in leaves and the pigment composition of individual subunits (PDB ID: 5XNM, (Su et al. 2017)).

**Supplementary Figure S2**

# Supplementary Figure S2. (A) Synopsis of the LHC composition for the genotypes utilized in this study. (B) Mapping of Lhcb genes across the *Arabidopsis* genome, using the TAIR chromosome map utility (http://www.arabidopsis.org/jsp/ChromosomeMap/tool.jsp).

**Supplementary Figure S3**

**Supplementary Figure S3. Composition of photosynthetic pigments in leaves from wild type and mutant lines.** Pigments were extracted from dark-adapted leaves with 85% acetone, then separated and quantified through HPLC. Carotenoid content was normalized to 100 molecules of Chl *a* + *b* and expressed as mean ± SD, n = 5 biologically independent samples. Values marked with different letters are significantly different from each other (ANOVA followed by Tukey’s post-hoc test at a significance level of P < 0.05).

The pigment composition of *koLHC* genotypes corresponds to the abundance of their pigment-protein complexes: the Chl *a*/*b* ratio (panel A) was lowest in *NoM* (3.28 *vs.* 3.51 in the wild type), highest in *koLhcb* (6.21) and intermediate in *koLHCII* (5.19). This is consistent with the lower Chl *b* content of monomeric Lhcbs *vs.* LHCII. LHCI complexes, which were unaffected in our genotypes, account for the residual Chl *b* content in *koLhcb*. The Chl/Car ratio (panel B) ranged from 3.1 to 3.7 depending on LHC content. HPLC analysis of carotenoid composition (panel C) revealed that β-carotene levels doubled in *koLhcb* compared to the LHCII-rich wild type and *NoM*. Meanwhile, *koLHCII* and *koLhcb* saw significant reductions in neoxanthin content by 60% and 80%, respectively, with a concurrent increase in violaxanthin, owing to the preferential location of neoxanthin in trimeric LHCII and absence in PSI-LHCI complexes (Schiphorst and Bassi 2020). In terms of leaf Chl content (panel D), there was a 25% reduction in *NoM* compared to the wild type, while *koLhcb* lines contained about 50% of the Chls content found in *koLHCII* and 25% relative to the wild type, suggesting that the growth rate per pigment unit was higher in the presence of monomeric antenna proteins.

**Supplementary Figure S4**

**Supplementary Figure S4.** **Characterization of the photosynthetic apparatus.** (A) The quantification of PSII / PSI ratio in wild type and mutant thylakoids was carried out using immunoblot analysis with antibodies targeted at specific gene products: the PSII core subunit PsbB (CP47) and the PSI core subunit (PsaA). Data were normalized to the corresponding wild type level. (B) The analysis employed antibodies specific to individual gene products, the PSII core subunit PsbB (CP47) and the PSI core subunit PsaA. The PSI/PSII ratio of *cutr1ac* was assessed by densitometric analysis of the nondenaturing PAGE (Figure S9B). (C) The functional antenna size of PSII was assessed in leaves by determining the rise time of Chl fluorescence in the presence of DCMU. The reciprocal of the time corresponding to two-thirds of the fluorescence rise (t_2/3_) served as a measure of the PSII antenna cross-section. (D) Representative Chl fluorescence rise kinetics, recorded in DCMU-treated leaves. Data are expressed as mean ± SD, n = 4 (panel A) or n = 12 (panel C) biologically independent samples. Values marked with different letters are significantly different from each other (ANOVA followed by Tukey’s post-hoc test at a significance level of P < 0.05).

**Supplementary Figure S5**

**Supplementary Figure S5. Growth curves of plants measured at different light intensities.** The total leaf area of plants cultivated at low light (LL, 120 µmol photons m^-2^ s^-1^) or high light (HL, 400 µmol photons m^-2^ s^-1^) was assessed using non-invasive image analysis. Values are expressed as mean ± SD; sample sizes are consistent with those in Figure 1C.

**Supplementary Figure S6**

**Supplementary Figure S6. Transmission electron micrographs of plastids in *koLhcb* leaf mesophyll cells.** Plants were dark-adapted for 2 hours before leaf harvesting. Samples were then fixed, embedded, and examined in thin sections at various magnifications. The chloroplasts of *koLhcb* plants were mostly devoid of grana; only in a few images did we detect the presence of sparse grana.

**Supplementary Figure S7**

**Supplementary Figure S7. Membrane macroorganization changes probed by circular dichroism.** (A) Circular dichroism (CD) spectra of thylakoid membranes isolated from wild type, *NoM*, *koLHCII*, and *koLhcb* genotypes were obtained after resuspending the membranes in stacking buffer, which contained 300 mM sorbitol and 5 mM MgCl_2_. (B) CD spectra of thylakoid membranes resuspended in unstacking buffer (devoid of sorbitol and MgCl_2_) were also recorded. The spectra presented are averages of three independent measurements, normalized to the absorbance at 675 nm.

Pigment circular dichroism (CD) spectroscopy is a sensitive non-destructive probe for the thylakoid membrane macroorganization (Garab 2016; Lambrev and Akhtar 2019). The CD spectra of granal thylakoid membranes typically show large non-conservative “psi”-type CD with characteristic bands in the chlorophyll Q_y_ region at 670 and 690 nm and in the carotenoid absorption region at 510 nm that have been associated with long-range interactions between pigments having a specific three-dimensional macroorganization (Garab and van Amerongen 2009). Previous studies on antenna mutants of *A. thaliana* have shown that specific PSII antenna deletions can bring about drastic changes in the psi-type CD spectra that correlated with changes in the membrane stacking as well as in the lateral macroorganization of the PSII-LHCII supercomplexes (Kovács et al. 2006; Tóth et al. 2016). Thus, the main positively signed psi-type band with a maximum at 690 nm that was primarily associated with the arrangement of PSII supercomplexes, was strongly diminished in the *koLhcb6* line of *A. thaliana* as well as in mutants with antisense suppression of Lhcb2 (Tóth et al. 2016). In line with this, our results show that the main Chl psi-type CD band at 690 nm had reduced amplitude in the *koLhcb* and *koLHCII* genotypes, to about 50-55% relative to WT (Figure S5A). Strong decrease in amplitude was also found for the negative CD band at 670 nm, which has been correlated with membrane stacking (Garab et al. 1991). Resuspension of the thylakoid membranes in hypotonic low-salt buffer resulted in a clear loss of the negative psi-type CD at 670 nm, consistent with membrane destacking (Supplementary Figure S5B). A much smaller effect was observed in the *koLhcb* and *koLHCII* membranes, which were evidently already in a partially destacked state. Thus, the CD spectra independently confirm that the loss of LHCII and of LHCII together with the monomeric Lhcb proteins results in marked changes in the membrane macroorganization and significant loss of membrane stacking.

In contrast, the psi-type CD of the *NoM* genotype was not only retained but had significantly larger amplitudes than the wild type. Thus, it could be conferred that trimeric LHCII or some of its subunits are largely responsible for the psi-type CD. On the other hand, the *NoM* membranes were more sensitive to resuspension in hypotonic Mg-free buffer than wild type, losing more than half of the psi-type CD intensity.

**Supplementary Figure S8**

**Supplementary Figure S8. Phosphorylation levels of Lhcb1/Lhcb2 in state 1- and state 2-adapted leaves.** Dark-adapted leaf discs were exposed to PSII light (200 µmol photons m^−2^ s^−1^) for 60 minutes to promote LHCII phosphorylation and the transition to state 2. Reference samples consisted of leaf discs that were dark-adapted for 2 hours. After treatment, samples were snap-frozen, and Lhcb1/Lhcb2 phosphorylation levels were quantified by immunotitration using α-Lhcb1-P and α-Lhcb2-P primary antibodies. Proteins corresponding to 0.8 µg of Chls for the wild type sample, and 2.4 µg of Chls for the mutant samples, were loaded for analysis. All samples were processed on the same SDS-PAGE slab gel.

**Supplementary Figure S9**

**Supplementary Figure S9. Changes in chloroplast ultrastructure in state 1- and state 2-adapted leaves.** Statistical analysis of average grana height in state 1 [n = 57 (wild type), 55 (*lowLHCII*), 58 (*Lhcb2-only*)] and state 2 [n = 58 (wild type), 60 (*lowLHCII*), 57 (*Lhcb2-only*)] is reported. All individual data points are shown; in the box plots, the boxes represent the interquartile areas, the horizontal lines indicate the medians, and the whiskers extend to the 5^th^ and 95^th^ percentiles. Values that exhibit significant differences (ANOVA followed by Tukey’s post-hoc test at a significance level of P < 0.05) are indicated with different letters. All experiments were independently repeated twice, yielding similar results.

**Supplementary Figure S10**

**Supplementary Figure S10. Biophysical characterization of mechanisms regulating energy transduction reactions in thylakoids.** (A) PSII repair efficiency. Detached leaves were exposed to EL for 3 hours (wild type, 1100 μmol photons m^−2^ s^−1^; *koLHCII*, 900 μmol photons m^−2^ s^−1^; *koLhcb*, 750 μmol photons m^−2^ s^−1^ – indicated by the yellow bar) at 4°C, which reduced the PSII maximal quantum yield (F_v_/F_m_) to approximately 30% of its initial value. Recovery of F_v_/F_m_ was subsequently assessed at 15 μmol photons m^−2^ s^−1^, 24°C (shown by the gray bar), favoring the PSII repair process (Mattoo et al. 1999). The wild type and *koLHCII* genotypes showed the same rate of PSII recovery. A similar dataset was published previously for the *NoM* genotype (Dall’Osto et al. 2020), showing no difference in the kinetic of recovery with respect to wild type plants. *koLhcb* healed its PSII activity however to a lower extent, suggesting that lateral heterogeneity between fully functional PSII in grana and PSII under repair in stroma-exposed margins might be necessary for a fully functional PSII repair cycle. (B) Relationship between pmfLET and ECSt in wild type and mutant lines during steady state illumination. We measured Chl fluorescence and the kinetic of electrochromic shift (ECS) on leaves, as a measure of electrons and protons transfer in the photosynthetic apparatus, respectively. The quantum yield of PSII photochemistry can be used to measure the electron released from PSII (LEF) in leaves under a different light. ECS kinetics can be used to estimate fluxes of protons through the thylakoids, upon steady-state actinic illumination (Cruz et al. 2005): ECS_t_, the total amplitude of the ECS signal upon light-dark transition, measures the light-driven protonmotive force (pmf) across the membrane, while the ECS decay lifetime (gH^+^) assesses the conductivity of the thylakoid ATPase to proton efflux. Under steady-state photosynthetic electron transport, the pmf produced by LEF alone can be estimated by the LEF/gH^+^ ratio, a parameter termed pmfLEF (Avenson et al. 2005). It turned out that the slope of the linear relationship of pmfLEF *vs.* ECS_t_ was proportional to the stoichiometry of electron *vs*. proton transfer. Figure 5B shows that wild type and mutant plants produced approximately the same extent of pmf that can be accounted for by LEF only, thus suggesting a similar CEF amplitude in all genotypes. Values are expressed as mean ± SD, n = 4. The experiments were repeated independently twice, yielding consistent results.

**Supplementary Figure S11**

**Supplementary Figure S11. Characterization of the *curt1ac* mutant.** (A) Transmission electron micrographs of plastids from leaf mesophyll cells in wild type and *curt1ac* lines. (B) Thylakoid pigment–protein complexes were separated by non-denaturing Deriphat-PAGE upon solubilization with 0.8% α-DM (dodecyl-D-maltopyranoside). Each lane was loaded with thylakoid proteins corresponding to 35 µg of chlorophylls. The composition of major bands is indicated, based on previous studies. (C) Densitometric analysis of selected thylakoid proteins from SDS-PAGE. Data of PSI core (PsaA) and Lhcb antenna subunits were normalized to OEC (PsbO), and expressed as a percentage of the corresponding wild type content. Data are shown as mean ± SD (n = 4). (D) Growth phenotypes under limiting and excess light conditions were examined. Plants were cultivated for 6 weeks under two light environments: 120 µmol photons m^−2^ s^−1^ at 23 °C, with an 8/16 hour light/dark cycle (LL, upper panel), and 400 µmol photons m^−2^ s^−1^ at 23 °C, with the same light/dark cycle (HL, lower panel). Data are expressed as mean ± SD, [n = 7 (wild type, *koLHCII* #17, *koLhcb* #9), 9 (*NoM, curt1ac*)]. Values marked with different letters are significantly different from each other (ANOVA followed by Tukey’s post-hoc test at a significance level of P < 0.05). (E) The total leaf area of plants grown under LL or HL conditions was evaluated through non-invasive image analysis. Values are expressed as mean ± SD, with sample sizes consistent with those in Figure 1C. (F,G) CD spectra of thylakoid membranes isolated from wild type and *curt1ac* were obtained after resuspending the membranes either in stacking buffer (F) or in unstacking buffer (G). The spectra presented are averages of three independent measurements, normalized to the absorbance at 675 nm. Membranes of the *curt1*ac line showed practically no psi-type CD in either buffer medium, despite the presence of appressed granal thylakoids, showing that membrane stacking is not sufficient to produce the characteristic psi-type CD and that the supramolecular organization of the pigment-protein complexes in the *curt1ac* mutant is altered severely.

**Supplementary Figure S12**

**curt1ac**

**Supplementary Figure S12. Decay-associated emission spectra (DAES) of thylakoid membranes isolated from both WT and mutants of *Arabidopsis* plants.** DAES were obtained through global lifetime analysis of fluorescence decays, recorded at room temperature with 440 nm excitation. Each spectrum represents an average from three independent measurements, with shaded areas indicating the standard error. DAES comparison between stacked (solid line) and unstacked (dashed line) membranes are presented, scaled such that their sum equals one at 680 nm. Note that only the symbols represent actual measured data points, whereas the lines are interpolated to guide the eye.

**Supplementary Figure S13**

**Supplementary Figure S13. Kinetic model of energy spillover.** The model consists of compartments representing PSI (bulk and red Chls) and PSII (one Antenna/RC compartment and two non-emissive radical pair compartments). The arrows represent transfer rate constants between compartments (ns^-1^). To simulate spillover, one fraction (β) of PSI/PSII is connected with rate constants *k_s_* and *k_−s_*. Fluorescence kinetics were simulated by solving the model and applying Gaussian emission spectra as follows: “PSI bulk”, 688 nm, Δ = 18 nm, “PSI red”, 720 nm, Δ = 32 nm, “PSII Ant/RC”, 682 nm, Δ = 18 nm.

**Supplementary Figure S14**

**Supplementary Figure S14. Kinetic model results without and with spillover.** (A,C,E) Population dynamics of PSI/PSII, decay-associated spectra (DAES) and average fluorescence lifetimes obtained from the simulated kinetics without spillover. (B,D,F) Population kinetics, DAES and average lifetimes for simulated spillover, *k_s_* = 12 ns^−1^, *k_−s_* = 8 ns^−1^, *β* = 0.8.

**Supplementary Figure S15**

**Supplementary Figure S15. Schematic diagram showing the influence of LHC trimers *vs*. monomers on grana formation.** The upper panel shows the grana cross-section measured in wild type, *koLHCII* and *lowLHCII* (from Figure 2K), with all individual data points shown, while the lower panel presents models of PSII-Lhcb organization in these genotypes. Since *koLHCII* and *lowLHCII* have the same biochemical antenna size but different Lhcb complement, these data indicate that monomeric Lhcb is approximately three times more effective at inducing membrane stacking than trimeric LHCII. Trimeric LHCII is shown in green, monomeric Lhcb in blue, and the PSII core complex in grey (PDB ID: 5XNM, (Su et al. 2017)).

**Supplementary Figure S16**

**Supplementary Figure S16. Statistical analysis of the extent of thylakoid stacking in wild type and antenna mutants *koLhcb2* and *koLhcb5.*** (u*pper panel*) The table reports the following parameters: grana height [n = 60], grana length [n = 60], and grana per chloroplast [n = 37 (wild type), 20 (*koLhcb2*), 13 (*koLhcb5*)]**.** Within each column, values marked with the same letter are not significantly different from each other (ANOVA followed by Tukey's post-hoc test at a significance level of P < 0.05). (*lower panels*) Micrographs of wild type, *koLhcb2* and *koLhcb5* chloroplasts. Plants grown under short-day conditions were dark-adapted for 2 hours before harvesting their leaves. The samples were then fixed, embedded, and examined in thin sections at various magnifications.

**Supplementary Figure S17**

**Supplementary Figure S17. Quantification of PsbR content.** (A) Immunoblotting was used to quantify the PsbR subunit in wild type and mutant thylakoids. The analysis employed antibodies targeting the PsbB (CP47) core subunit and PsbR. Thylakoid samples corresponding to 2.0, 4.0, and 6.0 μg of chlorophyll were loaded for each. (B) Results of the immunotitration are shown, with PsbR content normalized to PsbB core levels and expressed relative to the wild type content. Data are expressed as mean ± s.d., n = 3. Values marked with different letters are significantly different from each other (ANOVA followed by Tukey’s post-hoc test at a significance level of P < 0.05). In the case of genotypes that developed multiple reaction bands in the Western blot, only the darkest bands (i.e. those showing the same mobility as the signal detected in the wild-type sample) were used for immunotitration and densitometric analysis.

**Supplementary Figure S18**

**Supplementary Figure S18. Schematic diagram showing the approaches used to quantitatively analyze grana structure from electron microscopy data.** (A) Measurement of grana height and diameter in regular (left panels) and irregular grana (right panels). (B) Measurement of grana cross-sectional area normalized to total chloroplast area. (C) Measurement of the number of grana per chloroplast.

**Supplementary Table S1. Analysis of RT Chl fluorescence during photosynthesis.** Fluorescence was measured in dark-adapted plants that had been grown for 6 weeks under either low light (LL, 120 µmol photons m^−2^ s^−1^ at 23 °C) or high light (HL, 400 µmol photons m^−2^ s^−1^ at 23 °C), i.e. under the same conditions described in Figure 1. Before measurements, plants were illuminated for 20 min at the same irradiance used during growth to reach steady-state photosynthesis; then the PSII photosynthetic efficiency (Ф_PSII_) and the PSII redox state (qL) were determined in LL-grown (light-yellow background) and HL-grown (dark-yellow background) plants. Data are shown as means ± SD (n = 5). HL values that differ significantly from the corresponding LL values (Student’s *t* test, p < 0.05) are indicated with an asterisk. For each photosynthetic parameter, the last column reports the magnitude of the change in HL- *vs*. LL-acclimated plants. The increase in Ф_PSII_ observed in HL-grown *NoM* plants can be explained by a more extensive degradation of detached LHCII in this genotype upon HL acclimation compared with WT plants (*NoM* HL *vs*. LL, -59.7% % LHCII; WT, -18.7% % LHCII).

**Supplementary Table S2. Pigment content of PSII core complex and PSI-LHCI, isolated from wild type and mutant thylakoids**. Thylakoid membranes isolated from wild type and mutant plants were solubilized using 1.0 % β-DM, followed by the fractionation of pigment-protein complexes through sucrose gradients ultracentrifugation. Data are normalized to 100 Chl molecules and are expressed as mean ± SD (n = 4).

**Supplementary References**

**Avenson TJ, Cruz JA, Kanazawa A, and Kramer DM**. Regulating the proton budget of higher plant photosynthesis. Proc Natl Acad Sci U S A. 2005:**102**(27):9709–9713. https://doi.org/10.1073/pnas.0503952102

**Cruz JA, Avenson TJ, Kanazawa A, Takizawa K, Edwards GE, and Kramer DM**. Plasticity in light reactions of photosynthesis for energy production and photoprotection. . In. Journal of Experimental Botany. (J Exp Bot), pp. 395–406. https://doi.org/10.1093/jxb/eri022

**Dall’Osto L, Cazzaniga S, Bressan M, Paleček D, Židek K, Niyogi KK, Fleming GR, Zigmantas D, and Bassi R**. Two mechanisms for dissipation of excess light in monomeric and trimeric light-harvesting complexes. Nat Plants. 2017:**3**(April):17033. https://doi.org/10.1038/nplants.2017.33

**Dall’Osto L, Cazzaniga S, Zappone D, and Bassi R**. Monomeric light harvesting complexes enhance excitation energy transfer from LHCII to PSII and control their lateral spacing in thylakoids. Biochim Biophys Acta Bioenerg. 2020:**1861**(4):0–1. https://doi.org/10.1016/j.bbabio.2019.06.007

**Garab G**. Self-assembly and structural-functional flexibility of oxygenic photosynthetic machineries: Personal perspectives. Photosynth Res. 2016:**127**(1):131–150. https://doi.org/10.1007/s11120-015-0192-z

**Garab G and van Amerongen H**. Linear dichroism and circular dichroism in photosynthesis research. Photosynth Res. 2009:**101**(2–3):135–146. https://doi.org/10.1007/s11120-009-9424-4

**Garab G, Kieleczawa J, Sutherland JC, Bustamante C, and Hind G**. ORGANIZATION OF PIGMENT-PROTEIN COMPLEXES INTO MACRODOMAINS IN THE THYLAKOID MEMBRANES OF WILD-TYPE and CHLOROPHYLL fo-LESS MUTANT OF BARLEY AS REVEALED BY CIRCULAR DICHROISM. Photochem Photobiol. 1991:**54**(2):273–281.

**Guardini Z, Dall’Osto L, Gomez RL, Caferri R, Joliot P, and Bassi R**. Mapping light-harvesting and photoprotection responses in the Photosystem II antenna system of higher plants. Plant Physiol. 2025. https://doi.org/10.1093/plphys/kiaf588

**Kovács L, Damkjær J, Kereïche S, Ilioaia C, Ruban A V., Boekema EJ, Jansson S, and Horton P**. Lack of the light-harvesting complex CP24 affects the structure and function of the grana membranes of higher plant chloroplasts. Plant Cell. 2006:**18**(11):3106–3120. https://doi.org/10.1105/tpc.106.045641

**Lambrev PH and Akhtar P**. Macroorganisation and flexibility of thylakoid membranes. Biochemical Journal. 2019:**476**(20):2981–3018. https://doi.org/10.1042/BCJ20190080

**Malzahn A, Lowder L, and Qi Y**. Plant genome editing with TALEN and CRISPR. Cell Biosci. 2017:**7**(1):1–18. https://doi.org/10.1186/s13578-017-0148-4

**Mattoo AK, Giardi M-T, Raskind A, and Edelman M**. Dynamic metabolism of photosystem II reaction center proteins and pigments. Physiol Plant. 1999:**107**(4):454–461. https://doi.org/10.1034/J.1399-3054.1999.100412.X

**Ordon J, Bressan M, Kretschmer C, Dall’Osto L, Marillonnet S, Bassi R, and Stuttmann J**. Optimized Cas9 expression systems for highly efficient Arabidopsis genome editing facilitate isolation of complex alleles in a single generation. Funct Integr Genomics. 2020:**20**(1):151–162. https://doi.org/10.1007/s10142-019-00665-4

**Schiphorst C and Bassi R**. Chlorophyll-Xanthophyll Antenna Complexes: In Between Light Harvesting and Energy Dissipation. 2020:27–55. https://doi.org/10.1007/978-3-030-33397-3_3

**Su X, Ma J, Wei X, Cao P, Zhu D, Chang W, Liu Z, Zhang X, Li M, Ma J, et al.** Structure and assembly mechanism of plant C2S2M2-type PSII-LHCII supercomplex. Science (1979). 2017:**357**(6353):815–820. https://doi.org/10.1126/science.aan0327

**Tóth TN, Rai N, Solymosi K, Zsiros O, Schröder WP, Garab G, van Amerongen H, Horton P, and Kovács L**. Fingerprinting the macro-organisation of pigment--protein complexes in plant thylakoid membranes in vivo by circular-dichroism spectroscopy. Biochimica et Biophysica Acta (BBA)-Bioenergetics. 2016:**1857**(9):1479–1489.
