# Supplementary material for: Specific light-harvesting complexes mediate grana stacking and prevent energy spillover between photosystems in plant chloroplasts
Source: Plant Cell. 2026 Sep 1;38(9):koag256. doi: 10.1093/plcell/koag256 (PMC13564224; doi:10.1093/plcell/koag256)
Supplement: Supplementary file 1 [file koag256_supplementary_data.zip › statistics_figures.pdf]

Figure 1B

One Way Analysis of Variance

Group Name	N	Missing	Mean	Std Dev	SEM
Row 1 WT	11	0	0.498	0.0545	0.0164
Row 2 NoM	11	0	0.0475	0.0123	0.00370
Row 3 koLHCII #17	10	0	0.0554	0.0178	0.00564
Row 4 koLHCII #36	12	0	0.0601	0.0122	0.00353
Row 5 koLhcb #9	10	0	0.00220	0.00103	0.000327
Row 6 koLhcb #16	10	0	0.00290	0.000738	0.000233

Source of Variation	DF	SS	MS	F	P
Between Groups	5	1.986	0.397	645.130	<0.001
Residual	58	0.0357	0.000616		
Total	63	2.022			

The differences in the mean values among the treatment groups are greater than would be expected by chance; there is a statistically significant difference (P = <0.001).

Power of performed test with alpha = 0.050: 1.000

All Pairwise Multiple Comparison Procedures (Tukey Test):

Comparisons for factor:

Comparison	Diff of Means	p	q	P	P<0.050
Row 1 vs. Row 5	0.496	6	64.639	<0.001	Yes
Row 1 vs. Row 6	0.495	6	64.548	<0.001	Yes
Row 1 vs. Row 2	0.450	6	60.174	<0.001	Yes
Row 1 vs. Row 3	0.442	6	57.700	<0.001	Yes
Row 1 vs. Row 4	0.438	6	59.757	<0.001	Yes
Row 4 vs. Row 5	0.0579	6	7.705	<0.001	Yes
Row 4 vs. Row 6	0.0572	6	7.612	<0.001	Yes
Row 4 vs. Row 2	0.0125	6	1.712	0.830	No
Row 4 vs. Row 3	0.00468	6	0.623	0.998	Do Not Test
Row 3 vs. Row 5	0.0532	6	6.780	<0.001	Yes
Row 3 vs. Row 6	0.0525	6	6.691	<0.001	Yes
Row 3 vs. Row 2	0.00785	6	1.025	0.978	Do Not Test
Row 2 vs. Row 5	0.0453	6	5.915	0.001	Yes
Row 2 vs. Row 6	0.0446	6	5.824	0.002	Yes
Row 6 vs. Row 5	0.000700	6	0.0892	1.000	No

A result of "Do Not Test" occurs for a comparison when no significant difference is found between two means that enclose that comparison. For example, if you had four means sorted in order, and found no difference between means 4 vs. 2, then you would not test 4 vs. 3 and 3 vs. 2, but still test 4 vs. 1 and 3 vs. 1 (4 vs. 3 and 3 vs. 2 are enclosed by 4 vs. 2: 4 3 2 1). Note that not testing the enclosed means is a procedural rule, and a result of Do Not Test should be treated as if there is no significant difference between the means, even though one may appear to exist.

Figure 1C

One Way Analysis of Variance

Group Name	N	Missing	Mean	Std Dev	SEM
Row 1 WT	10	0	1.963	0.302	0.0956
Row 2 NoM	11	0	0.414	0.0607	0.0183
Row 3 koLHCII #17	11	0	0.655	0.171	0.0515
Row 4 koLHCII #36	11	0	0.551	0.135	0.0406
Row 5 koLhcb #9	9	0	0.0360	0.00545	0.00182
Row 6 koLhcb #16	10	0	0.0430	0.0123	0.00389

Source of Variation	DF	SS	MS	F	P
Between Groups	5	24.967	4.993	209.732	<0.001
Residual	56	1.333	0.0238		
Total	61	26.300			

The differences in the mean values among the treatment groups are greater than would be expected by chance; there is a statistically significant difference (P = <0.001).

Power of performed test with alpha = 0.050: 1.000

All Pairwise Multiple Comparison Procedures (Tukey Test):

Comparisons for factor:

Comparison	Diff of Means	p	q	P	P<0.050
Row 1 vs. Row 5	1.927	6	38.439	<0.001	Yes
Row 1 vs. Row 6	1.920	6	39.349	<0.001	Yes
Row 1 vs. Row 2	1.549	6	32.493	<0.001	Yes
Row 1 vs. Row 4	1.412	6	29.619	<0.001	Yes
Row 1 vs. Row 3	1.308	6	27.437	<0.001	Yes
Row 3 vs. Row 5	0.619	6	12.622	<0.001	Yes
Row 3 vs. Row 6	0.612	6	12.838	<0.001	Yes
Row 3 vs. Row 2	0.241	6	5.180	0.007	Yes
Row 3 vs. Row 4	0.104	6	2.235	0.614	No
Row 4 vs. Row 5	0.515	6	10.502	<0.001	Yes
Row 4 vs. Row 6	0.508	6	10.656	<0.001	Yes
Row 4 vs. Row 2	0.137	6	2.945	0.311	No
Row 2 vs. Row 5	0.378	6	7.708	<0.001	Yes
Row 2 vs. Row 6	0.371	6	7.782	<0.001	Yes
Row 6 vs. Row 5	0.00700	6	0.140	1.000	No

Figure 2H

One Way Analysis of Variance

Group Name	N	Missing	Mean	Std Dev	SEM
Row 1 WT	122	0	15.859	5.718	0.518
Row 2 NoM	124	0	15.244	5.193	0.466
Row 3 koLHCII	124	0	14.718	6.147	0.552
Row 4 koLhcb	127	0	16.352	5.771	0.512
Row 5 lowLHCII	132	0	13.351	5.602	0.488
Row 6 ch1	132	0	12.479	4.698	0.409
Row 7 ch1koLhcb5	122	0	13.254	5.022	0.455

Source of Variation	DF	SS	MS	F	P
Between Groups	6	1635.606	272.601	9.124	<0.001
Residual	876	26171.255	29.876		
Total	882	27806.860			

The differences in the mean values among the treatment groups are greater than would be expected by chance; there is a statistically significant difference (P = <0.001).

Power of performed test with alpha = 0.050: 1.000

All Pairwise Multiple Comparison Procedures (Tukey Test):

Comparisons for factor:

Comparison	Diff of Means	p	q	P	P<0.050
Row 4 vs. Row 6	3.873	7	8.062	<0.001	Yes
Row 4 vs. Row 7	3.099	7	6.324	<0.001	Yes
Row 4 vs. Row 5	3.002	7	6.248	<0.001	Yes
Row 4 vs. Row 3	1.634	7	3.349	0.212	No
Row 4 vs. Row 2	1.109	7	2.272	0.678	Do Not Test
Row 4 vs. Row 1	0.493	7	1.007	0.992	Do Not Test
Row 1 vs. Row 6	3.380	7	6.963	<0.001	Yes
Row 1 vs. Row 7	2.605	7	5.265	0.004	Yes
Row 1 vs. Row 5	2.508	7	5.168	0.005	Yes
Row 1 vs. Row 3	1.141	7	2.314	0.659	Do Not Test
Row 1 vs. Row 2	0.615	7	1.248	0.975	Do Not Test
Row 2 vs. Row 6	2.764	7	5.719	0.001	Yes
Row 2 vs. Row 7	1.990	7	4.038	0.065	No
Row 2 vs. Row 5	1.893	7	3.916	0.082	Do Not Test
Row 2 vs. Row 3	0.525	7	1.070	0.989	Do Not Test
Row 3 vs. Row 6	2.239	7	4.632	0.018	Yes
Row 3 vs. Row 7	1.465	7	2.972	0.352	Do Not Test
Row 3 vs. Row 5	1.368	7	2.830	0.414	Do Not Test
Row 5 vs. Row 6	0.871	7	1.832	0.854	No
Row 5 vs. Row 7	0.0970	7	0.200	1.000	Do Not Test
Row 7 vs. Row 6	0.774	7	1.596	0.920	Do Not Test

A result of "Do Not Test" occurs for a comparison when no significant difference is found between two means that enclose that comparison. For example, if you had four means sorted in order, and found no difference between means 4 vs. 2, then you would not test 4 vs. 3 and 3 vs. 2, but still test 4 vs. 1 and 3 vs. 1 (4 vs. 3 and 3 vs. 2 are enclosed by 4 vs. 2: 4 3 2 1). Note that not testing the enclosed means is a procedural rule, and a result of Do Not Test should be treated as if there is no significant difference between the means, even though one may appear to exist.

Figure 2I

One Way Analysis of Variance

Group Name	N	Missing	Mean	Std Dev	SEM
Row 1 WT	60	0	440.249	127.842	16.504
Row 2 NoM	60	0	519.918	184.135	23.772
Row 3 koLHCII	60	0	459.746	124.406	16.061
Row 4 koLhcb	7	0	649.722	229.067	86.579
Row 5 lowLHCII	60	0	448.480	95.793	12.367
Row 6 ch1	60	0	497.681	116.830	15.083
Row 7 ch1koLhcb5	60	0	493.497	144.359	18.637

Source of Variation	DF	SS	MS	F	P
Between Groups	6	505959.986	84326.664	4.485	<0.001
Residual	360	6768892.456	18802.479		
Total	366	7274852.442			

The differences in the mean values among the treatment groups are greater than would be expected by chance; there is a statistically significant difference (P = <0.001).

Power of performed test with alpha = 0.050: 0.947

All Pairwise Multiple Comparison Procedures (Tukey Test):

Comparisons for factor:

Comparison	Diff of Means	p	q	P	P<0.050
Row 4 vs. Row 1	209.473	7	5.409	0.003	Yes
Row 4 vs. Row 5	201.242	7	5.197	0.004	Yes
Row 4 vs. Row 3	189.976	7	4.906	0.009	Yes
Row 4 vs. Row 7	156.225	7	4.034	0.065	No
Row 4 vs. Row 6	152.041	7	3.926	0.081	Do Not Test
Row 4 vs. Row 2	129.804	7	3.352	0.211	Do Not Test
Row 2 vs. Row 1	79.669	7	4.500	0.025	Yes
Row 2 vs. Row 5	71.438	7	4.035	0.065	No
Row 2 vs. Row 3	60.172	7	3.399	0.197	Do Not Test
Row 2 vs. Row 7	26.421	7	1.492	0.941	Do Not Test
Row 2 vs. Row 6	22.237	7	1.256	0.974	Do Not Test
Row 6 vs. Row 1	57.432	7	3.244	0.247	No
Row 6 vs. Row 5	49.201	7	2.779	0.437	Do Not Test
Row 6 vs. Row 3	37.935	7	2.143	0.736	Do Not Test
Row 6 vs. Row 7	4.184	7	0.236	1.000	Do Not Test
Row 7 vs. Row 1	53.248	7	3.008	0.337	Do Not Test
Row 7 vs. Row 5	45.017	7	2.543	0.549	Do Not Test
Row 7 vs. Row 3	33.751	7	1.907	0.829	Do Not Test
Row 3 vs. Row 1	19.497	7	1.101	0.987	Do Not Test
Row 3 vs. Row 5	11.266	7	0.636	0.999	Do Not Test
Row 5 vs. Row 1	8.231	7	0.465	1.000	Do Not Test

A result of "Do Not Test" occurs for a comparison when no significant difference is found between two means that enclose that comparison. For example, if you had four means sorted in order, and found no difference between means 4 vs. 2, then you would not test 4 vs. 3 and 3 vs. 2, but still test 4 vs. 1 and 3 vs. 1 (4 vs. 3 and 3 vs. 2 are enclosed by 4 vs. 2: 4 3 2 1). Note that not testing the enclosed means is a procedural rule, and a result of Do Not Test should be treated as if there is no significant difference between the means, even though one may appear to exist.

Figure 2J

One Way Analysis of Variance

Group Name	N	Missing	Mean	Std Dev	SEM
Row 1 WT	30	0	36.967	5.143	0.939
Row 2 NoM	30	0	36.900	4.664	0.851
Row 3 koLHCII	30	0	35.800	7.322	1.337
Row 4 koLhcb	30	0	1.267	1.999	0.365
Row 5 lowLHCII	30	0	13.833	4.594	0.839
Row 6 ch1	30	0	15.167	5.038	0.920
Row 7 ch1koLhcb5	30	0	9.867	1.432	0.261

Source of Variation	DF	SS	MS	F	P
Between Groups	6	39732.267	6622.044	300.321	<0.001
Residual	203	4476.133	22.050		
Total	209	44208.400			

The differences in the mean values among the treatment groups are greater than would be expected by chance; there is a statistically significant difference (P = <0.001).

Power of performed test with alpha = 0.050: 1.000

All Pairwise Multiple Comparison Procedures (Tukey Test):

Comparisons for factor:

Comparison	Diff of Means	p	q	P	P<0.050
Row 1 vs. Row 4	35.700	7	41.641	<0.001	Yes
Row 1 vs. Row 7	27.100	7	31.610	<0.001	Yes
Row 1 vs. Row 5	23.133	7	26.983	<0.001	Yes
Row 1 vs. Row 6	21.800	7	25.428	<0.001	Yes
Row 1 vs. Row 3	1.167	7	1.361	0.962	No
Row 1 vs. Row 2	0.0667	7	0.0778	1.000	Do Not Test
Row 2 vs. Row 4	35.633	7	41.564	<0.001	Yes
Row 2 vs. Row 7	27.033	7	31.532	<0.001	Yes
Row 2 vs. Row 5	23.067	7	26.906	<0.001	Yes
Row 2 vs. Row 6	21.733	7	25.350	<0.001	Yes
Row 2 vs. Row 3	1.100	7	1.283	0.972	Do Not Test
Row 3 vs. Row 4	34.533	7	40.281	<0.001	Yes
Row 3 vs. Row 7	25.933	7	30.249	<0.001	Yes
Row 3 vs. Row 5	21.967	7	25.622	<0.001	Yes
Row 3 vs. Row 6	20.633	7	24.067	<0.001	Yes
Row 6 vs. Row 4	13.900	7	16.213	<0.001	Yes
Row 6 vs. Row 7	5.300	7	6.182	<0.001	Yes
Row 6 vs. Row 5	1.333	7	1.555	0.928	No
Row 5 vs. Row 4	12.567	7	14.658	<0.001	Yes
Row 5 vs. Row 7	3.967	7	4.627	0.018	Yes
Row 7 vs. Row 4	8.600	7	10.031	<0.001	Yes

A result of "Do Not Test" occurs for a comparison when no significant difference is found between two means that enclose that comparison. For example, if you had four means sorted in order, and found no difference between means 4 vs. 2, then you would not test 4 vs. 3 and 3 vs. 2, but still test 4 vs. 1 and 3 vs. 1 (4 vs. 3 and 3 vs. 2 are enclosed by 4 vs. 2: 4 3 2 1). Note that not testing the enclosed means is a procedural rule, and a result of Do Not Test should be treated as if there is no significant difference between the means, even though one may appear to exist.

Figure 2K

One Way Analysis of Variance

Group Name	N	Missing	Mean	Std Dev	SEM
Row 1 WT	41	0	17.772	4.704	0.735
Row 2 NoM	47	0	11.763	3.687	0.538
Row 3 koLHCII	50	0	11.574	2.855	0.404
Row 4 koLhcb	41	0	0.286	0.566	0.0884
Row 5 lowLHCII	47	0	3.802	1.401	0.204
Row 6 ch1	44	0	9.165	2.918	0.440
Row 7 ch1koLhcb5	44	0	4.124	2.177	0.328

Source of Variation	DF	SS	MS	F	P
Between Groups	6	9159.635	1526.606	181.442	<0.001
Residual	307	2583.019	8.414		
Total	313	11742.654			

The differences in the mean values among the treatment groups are greater than would be expected by chance; there is a statistically significant difference (P = <0.001).

Power of performed test with alpha = 0.050: 1.000

All Pairwise Multiple Comparison Procedures (Tukey Test):

Comparisons for factor:

Comparison	Diff of Means	p	q	P	P<0.050
Row 1 vs. Row 4	17.485	7	38.599	<0.001	Yes
Row 1 vs. Row 5	13.970	7	31.873	<0.001	Yes
Row 1 vs. Row 7	13.648	7	30.654	<0.001	Yes
Row 1 vs. Row 6	8.607	7	19.332	<0.001	Yes
Row 1 vs. Row 3	6.197	7	14.341	<0.001	Yes
Row 1 vs. Row 2	6.008	7	13.708	<0.001	Yes
Row 2 vs. Row 4	11.477	7	26.185	<0.001	Yes
Row 2 vs. Row 5	7.962	7	18.817	<0.001	Yes
Row 2 vs. Row 7	7.639	7	17.756	<0.001	Yes
Row 2 vs. Row 6	2.598	7	6.039	<0.001	Yes
Row 2 vs. Row 3	0.189	7	0.453	1.000	No
Row 3 vs. Row 4	11.288	7	26.121	<0.001	Yes
Row 3 vs. Row 5	7.773	7	18.653	<0.001	Yes
Row 3 vs. Row 7	7.450	7	17.573	<0.001	Yes
Row 3 vs. Row 6	2.409	7	5.683	0.001	Yes
Row 6 vs. Row 4	8.879	7	19.942	<0.001	Yes
Row 6 vs. Row 5	5.363	7	12.466	<0.001	Yes
Row 6 vs. Row 7	5.041	7	11.528	<0.001	Yes
Row 7 vs. Row 4	3.838	7	8.619	<0.001	Yes
Row 7 vs. Row 5	0.322	7	0.749	0.998	No
Row 5 vs. Row 4	3.515	7	8.020	<0.001	Yes

Figure 3

t-test

The result of the equal variance test indicates the likelihood that the two groups are sampled from populations with equal variances, but does not guarantee the equality or inequality of the two variances.

Group Name	N	Missing	Mean	Std Dev	SEM
Row 1 koLhcb complemented with Lhcb1 (30% than WT)	30	0	7.310	2.700	0.493
Row 2 koLHCII	30	0	15.320	2.960	0.540

Difference of means -8.010

Use the results of Welch's test, where equal variances are not assumed, if the equality of the population variances of the two groups is in doubt.

Equal Variances Assumed (Student's t-test):

t = -10.951 with 58 degrees of freedom.

95 percent two-tailed confidence interval for difference of means: -9.474 to -6.546

Two-tailed P-value = 9.637E-16

The difference in the mean values of the two groups is greater than would be expected by chance; there is a statistically significant difference between the input groups (P = <0.001).

Equal Variances Not Assumed (Welch's t-test):

t = -10.951 with 57.517 degrees of freedom.

95 percent two-tailed confidence interval for difference of means: -9.474 to -6.546

Two-tailed P-value = 1.077E-15

The difference in the mean values of the two groups is greater than would be expected by chance; there is a statistically significant difference between the input groups (P = <0.001).

t-test

The result of the equal variance test indicates the likelihood that the two groups are sampled from populations with equal variances, but does not guarantee the equality or inequality of the two variances.

Group Name	N	Missing	Mean	Std Dev	SEM
Row 1 koLHCII	30	0	8.630	2.100	0.383
Row 2 Lhcb2-only	30	0	15.320	2.960	0.540

Difference of means -6.690

Use the results of Welch's test, where equal variances are not assumed, if the equality of the population variances of the two groups is in doubt.

Equal Variances Assumed (Student's t-test):

t = -10.096 with 58 degrees of freedom.

95 percent two-tailed confidence interval for difference of means: -8.016 to -5.364

Two-tailed P-value = 2.173E-14

The difference in the mean values of the two groups is greater than would be expected by chance; there is a statistically significant difference between the input groups (P = <0.001).

Equal Variances Not Assumed (Welch's t-test):

t = -10.096 with 52.292 degrees of freedom.

95 percent two-tailed confidence interval for difference of means: -8.019 to -5.361

Two-tailed P-value = 6.997E-14

The difference in the mean values of the two groups is greater than would be expected by chance; there is a statistically significant difference between the input groups (P = <0.001).

t-test

The result of the equal variance test indicates the likelihood that the two groups are sampled from populations with equal variances, but does not guarantee the equality or inequality of the two variances.

Group Name	N	Missing	Mean	Std Dev	SEM
Row 1 koLhcb complemented with Lhcb1 (30% than WT)	30	0	7.310	2.700	0.493
Row 2 Lhcb2-only	30	0	8.630	2.100	0.383

Difference of means -1.320

Use the results of Welch's test, where equal variances are not assumed, if the equality of the population variances of the two groups is in doubt.

Equal Variances Assumed (Student's t-test):

t = -2.114 with 58 degrees of freedom.

95 percent two-tailed confidence interval for difference of means: -2.570 to -0.0699

Two-tailed P-value = 0.0389

The difference in the mean values of the two groups is greater than would be expected by chance; there is a statistically significant difference between the input groups (P = 0.039).

Equal Variances Not Assumed (Welch's t-test):

t = -2.114 with 54.686 degrees of freedom.

95 percent two-tailed confidence interval for difference of means: -2.572 to -0.0683

Two-tailed P-value = 0.0391

The difference in the mean values of the two groups is greater than would be expected by chance; there is a statistically significant difference between the input groups (P = 0.039).

t-test

The result of the equal variance test indicates the likelihood that the two groups are sampled from populations with equal variances, but does not guarantee the equality or inequality of the two variances.

Group Name	N	Missing	Mean	Std Dev	SEM
Row 1 NoM	30	0	14.140	4.080	0.745
Row 2 koLhcb complemented with Lhcb1 (120% than WT)	30	0	14.290	3.610	0.659

Difference of means -0.150

Use the results of Welch's test, where equal variances are not assumed, if the equality of the population variances of the two groups is in doubt.

Equal Variances Assumed (Student's t-test):

t = -0.151 with 58 degrees of freedom.

95 percent two-tailed confidence interval for difference of means: -2.141 to 1.841

Two-tailed P-value = 0.881

The difference in the mean values of the two groups is not great enough to reject the possibility that the difference is due to random sampling variability. There is not a statistically significant difference between the input groups (P = 0.881).

Equal Variances Not Assumed (Welch's t-test):

t = -0.151 with 57.152 degrees of freedom.

95 percent two-tailed confidence interval for difference of means: -2.142 to 1.842

Two-tailed P-value = 0.881

The difference in the mean values of the two groups is not great enough to reject the possibility that the difference is due to random sampling variability. There is not a statistically significant difference between the input groups (P = 0.881).

t-test

The result of the equal variance test indicates the likelihood that the two groups are sampled from populations with equal variances, but does not guarantee the equality or inequality of the two variances.

Group Name	N	Missing	Mean	Std Dev	SEM
Row 1 WT	30	0	18.490	4.560	0.833
Row 2 koLhcb complemented with Lhcb1 (120% than WT)	30	0	14.140	4.080	0.745

Difference of means 4.350

Use the results of Welch's test, where equal variances are not assumed, if the equality of the population variances of the two groups is in doubt.

Equal Variances Assumed (Student's t-test):

t = 3.894 with 58 degrees of freedom.

95 percent two-tailed confidence interval for difference of means: 2.114 to 6.586

Two-tailed P-value = 0.000258

The difference in the mean values of the two groups is greater than would be expected by chance; there is a statistically significant difference between the input groups (P = <0.001).

Equal Variances Not Assumed (Welch's t-test):

t = 3.894 with 57.297 degrees of freedom.

95 percent two-tailed confidence interval for difference of means: 2.113 to 6.587

Two-tailed P-value = 0.000260

The difference in the mean values of the two groups is greater than would be expected by chance; there is a statistically significant difference between the input groups (P = <0.001).

t-test

The result of the equal variance test indicates the likelihood that the two groups are sampled from populations with equal variances, but does not guarantee the equality or inequality of the two variances.

Group Name	N	Missing	Mean	Std Dev	SEM
Row 1 WT	30	0	18.490	4.560	0.833
Row 2 NoM	30	0	14.290	3.610	0.659

Difference of means 4.200

Use the results of Welch's test, where equal variances are not assumed, if the equality of the population variances of the two groups is in doubt.

Equal Variances Assumed (Student's t-test):

t = 3.955 with 58 degrees of freedom.

95 percent two-tailed confidence interval for difference of means: 2.074 to 6.326

Two-tailed P-value = 0.000211

The difference in the mean values of the two groups is greater than would be expected by chance; there is a statistically significant difference between the input groups (P = <0.001).

Equal Variances Not Assumed (Welch's t-test):

t = 3.955 with 55.099 degrees of freedom.

95 percent two-tailed confidence interval for difference of means: 2.072 to 6.328

Two-tailed P-value = 0.000220

The difference in the mean values of the two groups is greater than would be expected by chance; there is a statistically significant difference between the input groups (P = <0.001).

Figure 4

t-test

The result of the equal variance test indicates the likelihood that the two groups are sampled from populations with equal variances, but does not guarantee the equality or inequality of the two variances.

Group Name	N	Missing	Mean	Std Dev	SEM
Row 1 Lhcb2-only dark	60	0	741.629	794.693	102.594
Row 2 Lhcb2-only light	60	0	448.767	390.265	50.383

Difference of means 292.862

Use the results of Welch's test, where equal variances are not assumed, if the equality of the population variances of the two groups is in doubt.

Equal Variances Assumed (Student's t-test):

t = 2.562 with 118 degrees of freedom.

95 percent two-tailed confidence interval for difference of means: 66.520 to 519.203

Two-tailed P-value = 0.0117

The difference in the mean values of the two groups is greater than would be expected by chance; there is a statistically significant difference between the input groups (P = 0.012).

Equal Variances Not Assumed (Welch's t-test):

t = 2.562 with 85.894 degrees of freedom.

95 percent two-tailed confidence interval for difference of means: 65.640 to 520.083

Two-tailed P-value = 0.0121

The difference in the mean values of the two groups is greater than would be expected by chance; there is a statistically significant difference between the input groups (P = 0.012).

t-test

The result of the equal variance test indicates the likelihood that the two groups are sampled from populations with equal variances, but does not guarantee the equality or inequality of the two variances.

Group Name	N	Missing	Mean	Std Dev	SEM
Row 1 lowLHCII dark	58	0	304.222	119.181	15.649
Row 2 lowLHCII light	58	0	302.646	58.856	7.728

Difference of means 1.576

Use the results of Welch's test, where equal variances are not assumed, if the equality of the population variances of the two groups is in doubt.

Equal Variances Assumed (Student's t-test):

t = 0.0903 with 114 degrees of freedom.

95 percent two-tailed confidence interval for difference of means: -32.999 to 36.151

Two-tailed P-value = 0.928

The difference in the mean values of the two groups is not great enough to reject the possibility that the difference is due to random sampling variability. There is not a statistically significant difference between the input groups (P = 0.928).

Equal Variances Not Assumed (Welch's t-test):

t = 0.0903 with 83.241 degrees of freedom.

95 percent two-tailed confidence interval for difference of means: -33.137 to 36.288

Two-tailed P-value = 0.928

The difference in the mean values of the two groups is not great enough to reject the possibility that the difference is due to random sampling variability. There is not a statistically significant difference between the input groups (P = 0.928).

t-test

The result of the equal variance test indicates the likelihood that the two groups are sampled from populations with equal variances, but does not guarantee the equality or inequality of the two variances.

Group Name	N	Missing	Mean	Std Dev	SEM
Row 1 WT dark	55	0	463.737	96.154	12.965
Row 2 WT light	55	0	424.499	68.727	9.267

Difference of means 39.237

Use the results of Welch's test, where equal variances are not assumed, if the equality of the population variances of the two groups is in doubt.

Equal Variances Assumed (Student's t-test):

t = 2.462 with 108 degrees of freedom.

95 percent two-tailed confidence interval for difference of means: 7.648 to 70.827

Two-tailed P-value = 0.0154

The difference in the mean values of the two groups is greater than would be expected by chance; there is a statistically significant difference between the input groups (P = 0.015).

Equal Variances Not Assumed (Welch's t-test):

t = 2.462 with 97.755 degrees of freedom.

95 percent two-tailed confidence interval for difference of means: 7.610 to 70.864

Two-tailed P-value = 0.0156

The difference in the mean values of the two groups is greater than would be expected by chance; there is a statistically significant difference between the input groups (P = 0.016).
